# Symbiotic responses of *Lotus japonicus* to two isogenic lines of a mycorrhizal fungus differing in the presence/absence of an endobacterium

**DOI:** 10.1111/tpj.15578

**Published:** 2021-12-06

**Authors:** Francesco Venice, Matteo Chialva, Guido Domingo, Mara Novero, Andrea Carpentieri, Alessandra Salvioli di Fossalunga, Stefano Ghignone, Angela Amoresano, Candida Vannini, Luisa Lanfranco, Paola Bonfante

**Affiliations:** ^1^ Department of Life Sciences and Systems Biology University of Turin Turin Italy; ^2^ National Research Council (CNR) Institute for Sustainable Plant Protection (IPSP) Turin Italy; ^3^ Department of Biotechnology and Life Sciences University of Insubria Varese Italy; ^4^ Department of Chemical Sciences University of Naples Federico II Napoli Italy

**Keywords:** endobacteria, *Candidatus* Glomeribacter gigasporarum, arbuscular mycorrhizal fungus, *Lotus japonicus*, dual RNA‐seq, organelles, phenylpropanoid metabolism, phosphate transport / lipid biosysnthesis

## Abstract

As other arbuscular mycorrhizal fungi, *Gigaspora margarita* contains unculturable endobacteria in its cytoplasm. A cured fungal line has been obtained and showed it was capable of establishing a successful mycorrhizal colonization. However, previous OMICs and physiological analyses have demonstrated that the cured fungus is impaired in some functions during the pre‐symbiotic phase, leading to a lower respiration activity, lower ATP, and antioxidant production. Here, by combining deep dual‐mRNA sequencing and proteomics applied to *Lotus japonicus* roots colonized by the fungal line with bacteria (B+) and by the cured line (B−), we tested the hypothesis that *L. japonicus* (i) activates its symbiotic pathways irrespective of the presence or absence of the endobacterium, but (ii) perceives the two fungal lines as different physiological entities. Morphological observations confirmed the absence of clear endobacteria‐dependent changes in the mycorrhizal phenotype of *L. japonicus*, while transcript and proteomic datasets revealed activation of the most important symbiotic pathways. They included the iconic nutrient transport and some less‐investigated pathways, such as phenylpropanoid biosynthesis. However, significant differences between the mycorrhizal B+/B− plants emerged in the respiratory pathways and lipid biosynthesis. In both cases, the roots colonized by the cured line revealed a reduced capacity to activate genes involved in antioxidant metabolism, as well as the early biosynthetic steps of the symbiotic lipids, which are directed towards the fungus. Similar to its pre‐symbiotic phase, the intraradical fungus revealed transcripts related to mitochondrial activity, which were downregulated in the cured line, as well as perturbation in lipid biosynthesis.

## Significance Statement

Using two isogenic lines of *Gigaspora margarita*, one containing and one lacking an endobacterium, for the first time we demonstrate that an endosymbiont hosted within the cytoplasm of an arbuscular mycorrhizal fungus has an impact on the regulatory networks of the plant host. OMICs analyses revealed that *Lotus japonicus* perceives the physiological differences between the two fungal isogenic lines; the absence of the bacterium leads to perturbation in mycorrhiza‐relevant pathways, which are associated with plant mitochondria and plastids.

## INTRODUCTION

Arbuscular mycorrhizal fungi (AMF) are among the most widespread fungi, as they are associated with more than 72% of terrestrial plants, including many relevant crops with which they form the AM symbiosis. AMF, which are obligate biotrophs, belong to the Glomeromycotina subphylum (Spatafora et al., 2016), which is an ancient clade of early diverging fungi, related to the Mucoromycota. As beneficial members of plant microbiota (Bonfante et al., 2019), AMF contribute to the uptake of nutrients from soil, increasing plant growth and conferring resistance to environmental stresses. The cellular and molecular mechanisms, which are required to establish a successful symbiosis between plant and fungal partners have been largely investigated thanks to the development of sequencing platforms, molecular techniques, and the use of plant mutants affected in their colonization capacities (Gutjahr and Parniske, 2013; Lanfranco et al., 2018). Many studies have therefore focused on local and systemic transcriptomic and proteomic changes in rice, maize, wheat, barrel clover, and tomato plants, which, as important crops and/or model plants, are among the more deeply investigated host plants (Genre et al., 2020 for a review). The sequencing of the *Rhizophagus irregularis* genome (Chen et al., 2018; Lin et al., 2014; Tisserant et al., 2013), of other Glomerales (*Rhizophagus clarus*, *Rhizophagus cerebriforme*, and *Rhizophagus diaphanous*; Morin et al., 2019) and Diversisporales species (*Diversispora* and *Gigaspora*; Morin et al., 2019; Sun et al., 2019; Venice et al., 2020), with the addition of several AMF transcriptomes (Salvioli et al., 2016; Tang et al., 2016), has also offered the novel opportunity to detect the fungal transcripts among the dominant reads of plant origin. However, our current knowledge remains plant‐centric, as many of the fungal genes expressed during the symbiotic phase are not annotated (Morin et al., 2019; Venice et al., 2020). The genome sequencing of some *Rhizophagus* species and isolates has revealed that only a core of genes is conserved, while a large part is isolate‐specific (Chen et al., 2018). These genomic data could give a mechanistic explanation for transcriptomic data revealing that genetically related AMF may have a different impact on the same host, as Mateus et al. (2019) demonstrated by studying the transcriptomic profiles of cassava upon colonization by two *R. irregularis* isolates. However, due to the huge genome variability occurring in the sequenced *R. irregularis* isolates (Morin et al., 2019), the fungal determinants that drive the plant to activate different responses remain unknown.

In this context and in the more general scenario of plant‐microbiota interactions, we used OMICs tools to investigate the responses of *Lotus japonicus* to *Gigaspora margarita*. The isolate BEG34 contains an endobacterium, *Candidatus* Glomeribacter gigasporarum (*Ca*Gg), which has a relevant impact on the fungal physiology (Salvioli et al., 2016) and a cured line with verified mycorrhizal capacities is available (Lumini et al., 2007). This cured line does not have a clear impact on the plant phenotype, differently from a virus‐cured line of a *Glomus* sp. strain, which stimulated plant growth (Ikeda et al., 2012). The two *G*. *margarita* lines are therefore isogenic with the exception of the presence/absence of *Ca*Gg, which has a reduced genome at about 1.9 Mb (Ghignone et al., 2012) versus the 770 Mb of the fungal host (Venice et al., 2020). By combining deep dual‐RNA sequencing and proteomics applied to *L. japonicus* roots colonized by the fungal line with bacteria (B+) and by the cured line (B−), we wanted to test the hypothesis that (i) *L. japonicus* activates its symbiotic pathways irrespective of the presence or the absence of the endobacterium inside *G. margarita*, but (ii) it perceives the two lines as different genetic and physiological entities. We reasoned that the experiments could also provide a further descriptive insight to *L. japonicus* responses to *G. margarita*, as after the pioneering description done by using microarray (Guether et al., 2009), reports on *L. japonicus* are limited to its association with *R. irregularis* (Handa et al., 2015; Sugimura and Saito, 2017), a mycorrhizal fungus that is phylogenetically far from *Gigaspora*.

As AMF mutants remain unavailable, the two isogenic lines, exclusively differing in a component of their meta‐genome (the endobacterium), offer an unprecedented tool to investigate the specificity of the plant response to its intracellular microbiota. Our results demonstrate how a host plant may not only perceive the presence of a mycorrhizal fungus, but also modulate its response depending on whether or not the fungus hosts an endobacterium.

## RESULTS AND DISCUSSION

### Mycorrhizal fungus more than the endobacterium is the driving factor that modulates the transcriptomic and proteomic profile of *Lotus japonicus* roots

**Figure 1:**
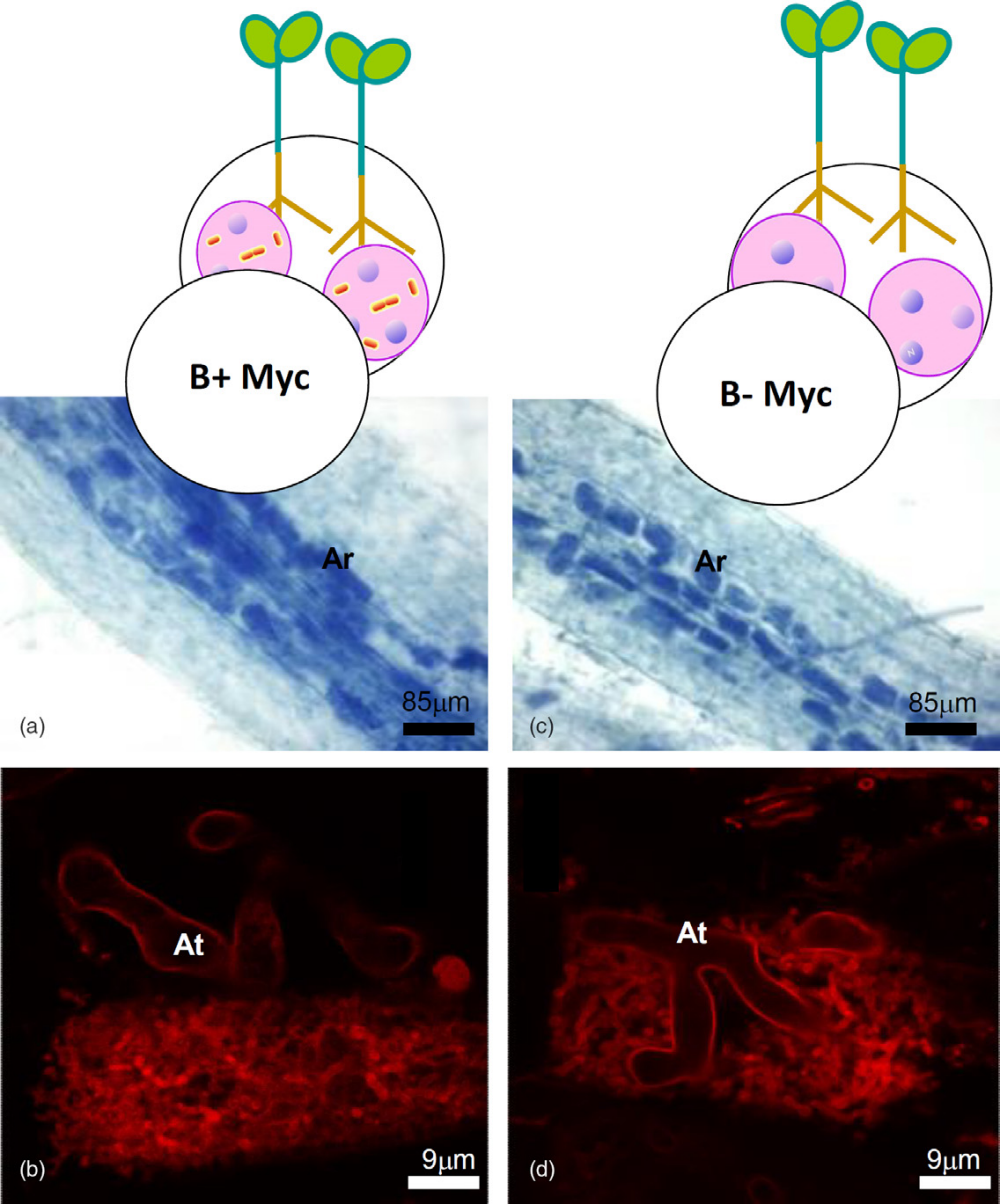
Mycorrhizal phenotype of *Lotus japonicus* roots colonized by the AM fungus *Gigaspora margarita* containing (B+) or not (B–) the endobacterium *Candidatus* Glomeribacter gigasporarum. (a,b) Roots mycorrhized by B+ fungus; (c,d) roots mycorrhized by B– fungus; (a,c) mycorrhizal roots stained with cotton blue to evaluate the intraradical fungal colonization. Fungal structures are stained dark blue. (b,d) Details of two arbuscules stained with wheat germ agglutinin conjugated with the fluorescent dye tetramethylrhodamine. Ar, arbuscules; At, arbuscule main trunks.

*Lotus japonicu*s seedlings mycorrhized by *G. margarita* with or without its endobacterium *Ca*Gg, were grown using the “sandwich” method, which guarantees a semi‐sterile condition (Novero et al., 2002), and sampled 4 weeks after inoculation. The mycorrhizal phenotype was similar in both the mycorrhizal samples (B+ Myc and B− Myc, see Figure 1), mirroring the colonization success of the B– fungal line (Lumini et al., 2007). No fungal structure was present in the non‐inoculated plants (NoMyc). The arbuscule phenotype was also unaffected by the bacterial presence/absence (Figure 1) as well as the general organization of the arbusculated plant cells, when seen using transmission electron microscopy (Figure S1).

To unravel whether *G. margarita* had an impact on the plant host, notwithstanding the absence of a clear phenotype, deep RNA‐sequencing (RNA‐seq) was performed on the B+ Myc, and B− Myc *L. japonicus* roots, using the NoMyc plants as controls. In total, nine libraries were obtained (three conditions with three biological replicates each). Sequencing yielded ~54 to ~95 million read pairs per library (Table S1). Alignment on the *L. japonicus* reference transcriptome (CDS, v.3.0) led to a mean mapping rate of 71.13%. Afterwards, reads counts were summarized at gene level, low‐count genes were then removed (see Experimental procedures section), and 71.56% of the plant genes used for subsequent analyses (26 557 out of 38 506 total genes). No fungal reads were detected in the NoMyc samples.

**Figure 2:**
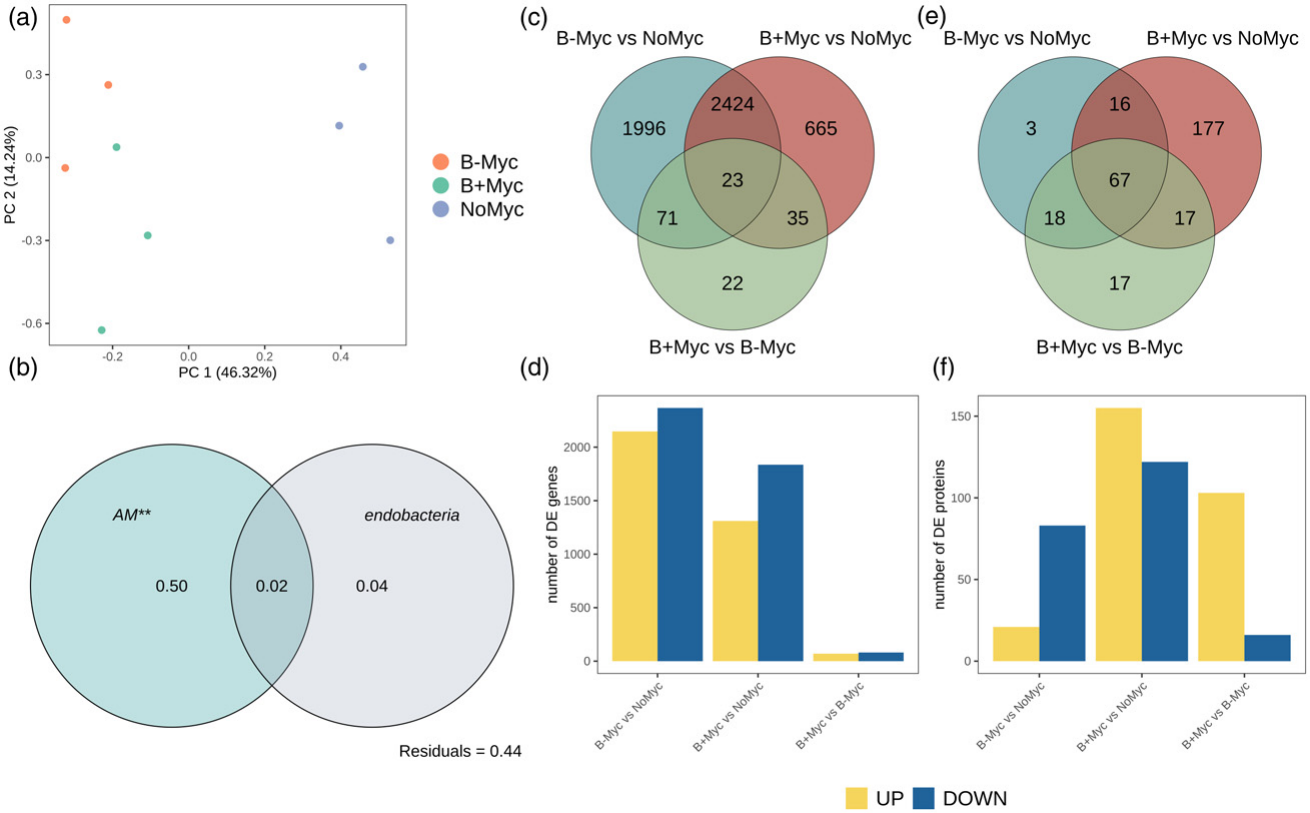
Multivariate transcriptome analysis and differential genes/proteins expression statistics of *Lotus japonicus* roots colonized by *Gigaspora margarita* (B+ and B− lines) and non‐mycorrhizal controls (NoMyc). (a) Principal components analysis (PCA) of deep‐sequencing libraries performed on normalized counts. (b) Partitioning of the global variance by each of the experimental factor considered, performed on whole transcriptomes according to variance partitioning analysis. Numbers within the Venn diagram represents the portion of explained variance by each factor alone. Statistical significance of single factors was tested on the redundancy analysis model using permutational ANOVA (999 permutations; *P < 0.05; **P < 0.005; *** *P* < 0.001). (c,d) Differential gene expression analysis; (e,f) differential protein expression analysis; (c,e) Venn diagrams showing the similarities of transcript/protein in the three investigated contrasts. (d,f) Bar plots showing the amount of upregulated (yellow) and downregulated (blue) transcripts and proteins in each contrast.

First, we applied variance partitioning analysis (VPA) coupled with principal components analysis (Figure 2a) to identify major sources of variation in the plant transcriptome. The principal components plot revealed two main clusters discriminated by the first principal component (PC1, *x*‐axis): the first grouping all the mycorrhized samples, the second the non‐mycorrhized ones. Even if overlapping, B− Myc and B+ Myc samples were partially discriminated by the second principal component (PC2, *y*‐axis). Accordingly, VPA showed that the largest portion of variance was explained by the presence of the AMF (50% variance explained, *P* < 0.01) while *Ca*Gg presence only explained a low amount of the transcriptome variance (4% variance explained, *P* > 0.05), while a large portion (44%) remained unexplained (Figure 2b).

The plant differentially expressed genes (DEGs) were identified (Data S1), considering the three contrasts according to the multifactorial set‐up, i.e., B− Myc versus NoMyc, B+ Myc versus NoMyc, and B+ Myc versus B− Myc. Figure S2 illustrates the experimental setup highlighting the three contrasts analyzed. While the first two comparisons detect *L. japonicus* responses to the mycorrhizal isogenic lines, the third comparison allows the detection of the specific impact of the presence/absence of the endobacterium. The highest number of DEGs was observed in B− Myc versus NoMyc contrast (4514 transcripts) followed by B+ Myc versus NoMyc (3147 transcripts) with a consistent portion shared between these two contrasts (2447 transcripts) (Figure 2c). The data reveal that the fungus on the whole modulates a relevant number of plant genes (5214), a figure that is considerably higher than the 3641 DEGs identified by Handa et al. (2015) by using the couple *L. japonicus/R. irregularis*. The presence/absence of the fungal endobacterium has a more limited impact with the differential expression of 151 genes (B+ Myc versus B− Myc). Both B− Myc versus NoMyc and B+ Myc versus NoMyc contrasts showed a prevalence of downregulated genes (Figure 2d). Twenty‐two genes were exclusive of the direct comparison B+ Myc versus B− Myc. The dual RNA‐seq approach also revealed fungal transcripts, which represented 1.75% of the whole reads; they identified 39.89% of the fungal genes (10 612 out of 26 603 predicted). No bacterial genes were detected in the B+ Myc samples. The analysis of fungal transcripts during the symbiotic phase of the B+ and B− *G. margarita* isogenic lines revealed that the presence of endobacteria led to a clear separation in the expression pattern of the two lines (Figure S3), with the modulation of 152 genes (Data S2). Of these, 119 were upregulated in the B+ fungus, compared with the cured line.

As a further step, total soluble protein extraction and proteome analysis using iTRAQ was performed on roots of B+ Myc, B− Myc and NoMyc plants. In total, 374 proteins were identified using the *L. japonicus* reference proteome (LotusBase) with a false discovery rate (FDR) <0.01. A total of 298 differentially expressed proteins (DEPs) were identified in Myc versus NoMyc roots, 277 of which in B+ Myc versus NoMyc and 104 in in B− Myc versus NoMyc (Data S3). Among them, 194 were B+ Myc‐specific and 21 B− Myc ‐specific (Figure 2e,f); 111 of the 194 B+ Myc‐specific DEPs were up‐ and 83 downregulated, while looking at the B− Myc ‐specific DEPs, six were up‐ and 15 downregulated. Among the shared DEPs, 48 were similarly regulated in B+ Myc/NoMyc and B− Myc /NoMyc comparisons, whereas 35 had diverging regulations, with 17 proteins exclusive of the direct comparison between the two lines of mycorrhizal plants.

In conclusion, as expected, mycorrhization was the main modulator of *L. japonicus* transcriptome and proteome reprogramming both in terms of DEGs and the DEP number (Figure 2c–f) and transcriptome variance explained (Figure 2b). By contrast, the direct comparison between B+ Myc and B− Myc plants generated a lower number of DEGs and DEPs, while relevant differences could be appreciated when B+ Myc and B− Myc plants were compared with NoMyc separately. Both the approaches demonstrate that *G. margarita* differentially modulates the *L. japonicus* gene and protein expression in the presence/absence of endobacteria. This latter feature also affects the fungal transcripts during the symbiotic intraradical phase. Based on this, we first focused our attention on the two comparisons between mycorrhizal plants (both B+ Myc and B− Myc) and the uninoculated control plants (NoMyc). The analysis revealed the activation of the most common symbiotic pathways, but with fine differences in some of them, which will be described in detail in the next paragraphs (phosphate transport, phenylpropanoid, and lipid metabolism). The other datasets are commented on in Appendix S1. As a second step, we focused on DEGs and DEPs, which emerged from the direct comparison between B+ Myc and B− Myc plants (Figure S2; Figure 2).

### *Lotus japonicus* activates AM‐induced symbiotic pathways, irrespective of the endobacterium

**Figure 3:**
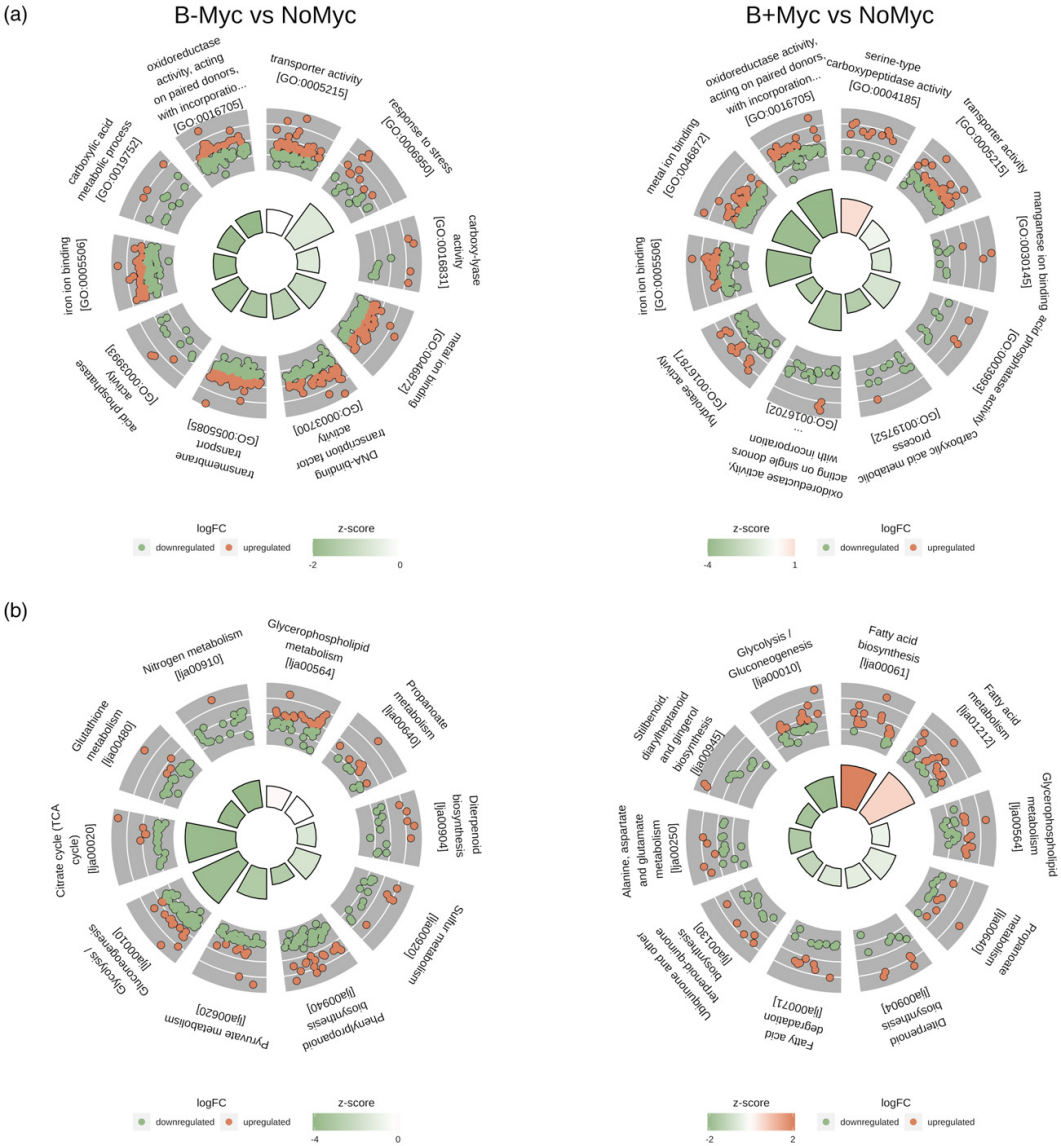
Ten top functional categories enriched (*P* < 0.05) among DEGs in *Gigaspora margarita* (B+ and B– isogenic lines) mycorrhized *Lotus japonicus* roots versus non‐mycorrhized controls (NoMyc). (a) Gene Ontology functional categories; (b) KEGG pathway. Each section of the circle plot represents an enriched term or pathway. Inner bar plot plots the –log_10_ of the adjusted *P*‐value for each enriched term and the color shows the *z*‐score value, indicating global upregulation (if >0) or downregulation (if <0) of genes within each category. Detailed log_2_fold‐change (log_2_FC) value of each gene within each category is plotted as a dot plot in the outer circle (upregulated in red and downregulated in green). The first 10 enriched categories were plotted clockwise by decreasing *z*‐score and adjusted *P*‐value.

**Figure 4:**
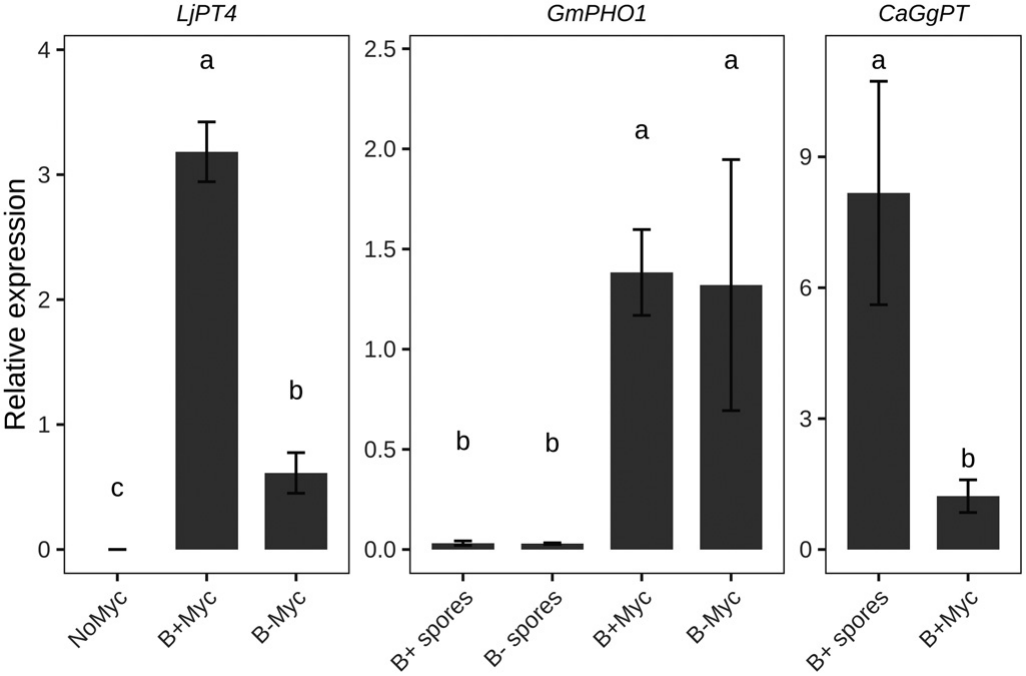
Expression of symbiosis‐related phosphate transporters across the tripartite symbiosis between *Lotus japonicus* plant, the arbuscular mycorrhizal fungus *Gigaspora margarita* and its endobacterium *Candidatus* Glomeribacter gigasporarum (*Ca*Gg), measured using real‐time quantitative polymerase chain reaction. Germinated spores were used as control condition for arbuscular mycorrhizal fungus and *Ca*Gg. Relative expression values were normalized on plant ubiquitin 10, the fungal elongation factor 1α and the bacterial 23S rRNA gene for *L. japonicus*, *G. margarita*, and *Ca*Gg, respectively. Bars represent the mean normalized expression value ± standard error. Letters indicate statistically supported differences according to the Tukey’s post‐hoc test (*P* < 0.05) after ANOVA (*P* < 0.05).

We searched for enriched functional categories in B− Myc versus NoMyc and B+ Myc versus NoMyc DEGs using Gene Ontology (GO) and KEGG (Kyoto Encyclopedia of Genes and Genomes) functional databases. Twenty‐one and 26 GO functional categories were enriched in the two comparisons, respectively (Data S4, Figure 3a). Most of the enriched GO categories were shared by B+/B− Myc versus NoMyc contrasts (13 terms), irrespective of the endobacterium, and included many of the known plant responses to AMF, i.e., DEGs involved in membrane trafficking, nutrients transport, iron ion binding, and phosphate metabolism. In particular, in the “transporter activity” category (GO:0005215), we found several genes coding for transporters of nitrate, potassium, boron, sulfate, molybdenum, zinc, and iron; the expression of genes related to this category had a balance between up‐ and downregulated genes in both the contrasts. In particular, we found that the mycorrhiza‐induced phosphate transporter 4 (LjPt4, Lj1g3v0948470) and ammonium transporter 2 (LjAMT2, Lj0g3v0115479), which are both well‐characterized AM symbiotic markers for *L. japonicus* (Guether et al., 2009; Volpe et al., 2016), were strongly upregulated upon symbiosis with both fungal lines. To understand this result better, we examined the expression of the phosphate transporter of all the partners by using real‐time quantitative polymerase chain reaction (qPCR). The experiment confirmed that *LjPT4* is upregulated in all the mycorrhizal *Lotus* plants, but its expression in B+ Myc is four times higher than in B− Myc (Figure 4). This does not seem to be related to the functionality of the fungal PHO1 where a phosphate transporter (PT) is directly involved in the P uptake from the soil and in its transfer to the plant at the arbuscule interface (Venice et al., 2020). Indeed, the expression of PHO1 in both the fungal lines was the same (Figure 4), even if the intraradical abundance of the fungus B– was slightly lower, as indicated by the expression of the housekeeping elongation factor gene (Figure S4). Lastly, we investigated the PT activity in the endobacterium, which fully depends on its fungal host for mineral nutrition (Ghignone et al., 2012). Interestingly, the expression level of the *Ca*Gg PT was higher in germinating spores when compared with the symbiotic mycelium (Figure 4), despite the lower abundance of endobacteria (Figure S4). These data suggest that the endobacterium does not represent a relevant cost for the symbiotic hyphae.

On the whole, the experiment suggests that the higher P content found in B+ Myc plants (Salvioli et al., 2016) potentially depends on the higher expression of the plant symbiotic transporter when the fungal partner contains the endobacterium.

KEGG pathways enrichment of DEGs showed 17 and 26 over‐represented pathways ID in B− Myc versus NoMyc and B+ Myc versus NoMyc, respectively (Data S4, Figure 3b). The vast majority of enriched pathways were shared between the two contrasts (16 categories) and included pathways already known to be modulated under AM symbiosis and in agreement with GO enriched terms. These pathways included “Glutathione metabolism” (lja00480), “Diterpenoid metabolism” (lja00904), “Nitrogen metabolism” (lja00910), “Sulfur metabolism” (lja00920), “Biosynthesis of secondary metabolites” (lja01110), and “Biosynthesis of amino acids” (lja01230).

The proteome dataset revealed 69 and 44 GO categories, as well as 17 and 15 KEGG pathways enriched in B+ Myc versus NoMyc and B− Myc versus NoMyc contrasts respectively (Data S5). Enriched pathways were involved in primary metabolisms such as “Carbon metabolism” (lja01200), “Pentose phosphate pathway” (lja00030) or “Glycolysis/Gluconeogenesis” (lja00010) (Figure S5). Moreover, pathways, which are considered as hallmarks of AM symbiosis emerged (Figure S5B): “Biosynthesis of amino acids” (lja01230), “Nitrogen metabolism” (lja00910), and “Glutathione metabolism” (lja00480). Similarly, we found GO enriched terms involved in defense response (GO:0006952), response to biotic stimulus (GO:0009607), and peroxidase activity (GO:0004601). Details on AM‐related categories enriched only in B+ Myc versus NoMyc are provided in Appendix S1.

In conclusion, GO and KEGG enrichment analyses revealed that many functional symbiosis‐related categories were activated in *L. japonicus* roots when colonized by *G. margarita*, irrespective of the presence/absence of its endobacterium. Many of these gene categories have already been described as AM‐responsive not only in *Lotus* (Sugimura and Saito, 2017) but also in other plants, from tomato and rice to wheat and *Medicago* (Fiorilli et al., 2018; Li et al., 2019; Song et al., 2015; Tian et al., 2019). These results on the one hand are confirmatory, showing that *G. margarita* can induce plant molecular responses as other more performant AM species, i.e. *Rhizophagus irregularis* (Sugimura and Saito, 2017). The B– fungal line revealed similar capacities to activate the mycorrhizal responses, confirming our previous morphological results (Lumini et al., 2007). However, as revealed by the expression of *LjPT4*, and by some KEGG gene and protein profiles there are relevant differences in *L. japonicus* responses when elicited by the two isogenic lines of a mycorrhizal fungus. They include fatty acid (FA) biosynthesis and metabolism, which were mostly upregulated in B+ Myc, and glutathione metabolism, which was mostly downregulated in B− Myc.

### Both *Gigaspora margarita* lines activate plant responses to abiotic and biotic stress, even if with slight differential patterns

In addition to the nutritional benefits, it is known that AM colonization elicits plant pathways, which help to respond to abiotic stress (Lenoir et al., 2016) and acts as a protective defense priming before a potential pathogen attack (Fiorilli et al., 2018; Martinez‐Medina et al., 2016; Vannini et al., 2021), a mechanism that involves the plant immune system (Zhou and Zhang, 2020). We therefore checked whether the two fungal lines could also elicit differential stress and defense responses.

The “response to stress” category (GO:0006950) emerged as over‐represented and enriched in the B− Myc versus NoMyc: it was represented by 23 transcripts, mostly downregulated (*z*‐score <0, Figure 3a). In the B+ Myc versus NoMyc comparison, 14 genes belonging to this category were present, displaying a comparable expression trend (Appendix S1). Among them, a number of universal stress proteins (USPs) were strongly induced upon *G. margarita* colonization being more abundant in the B– Myc plants versus B+ Myc ones (five versus three genes, respectively). These proteins, which are highly conserved through all kingdoms of life and highly expressed under diverse environmental constraints, have not been extensively characterized, but they probably act as molecular chaperones. In *A. thaliana*, they have been involved in the interaction with thioredoxin‐h1, a reactive oxygen species (ROS) scavenger protein, and modulating ROS concentration (Chi et al., 2019).

To understand to what extent *L. japonicus* genes can also be involved in the plant defense response, we visualized the plant–pathogen interaction KEGG map (ko:04626, Figure S6). The map revealed that the plant‐triggered immunity (PTI) was elicited in both fungal lines, when compared with the control, with a similar upregulation of calcium signaling‐related proteins including plasma membrane‐located cyclic nucleotide‐gated channels, which converged in the upregulation of respiratory burst oxidase homolog genes (Lj2g3v2082320, Lj5g3v1497820) and in the activation of the WRKY transcription factor 29 (WRKY29, Lj4g3v2990170). This last is an essential component of defense responses against pathogens within the mitogen‐activated protein kinase signaling pathway (Jiang et al., 2017).

Searching for responses, which seemed to be endobacterial‐dependent, we found a finely tuned regulation of plant genes, which are often associated to bacterial exposure (Teixeira et al., 2019). The *L. japonicus* genome possesses 25 pathogenesis‐related proteins (PRs), which are expressed downstream of the PTI pathway. We detected seven PR genes in the B− Myc versus NoMyc comparison (five up‐ and two downregulated): three of them were shared with the B+ Myc plants and showed the same expression pattern (two down‐ and one upregulated). In the same context, BAK1 (the BRASSINOSTEROID INSENSITIVE 1‐associated receptor kinase 1‐like, Lj5g3v1749340) emerged as uniquely upregulated in the B+ Myc versus NoMyc contrast. BAK1 is a well‐characterized receptor, which together with FLS2 activates PAMP perception, initiating the downstream PTI pathway (Chinchilla et al., 2007).

Proteomics confirmed the regulation of several proteins involved in plant defense response in both B+ Myc and B− Myc roots, as well as the impact on the induction of such proteins depending on the presence/absence of the endobacterium. Two PR10 (Lj0g3v0348509, Lj6g3v1513720) were upregulated in the direct B+ Myc versus B− Myc comparison, as well as the MLP‐like protein 28‐like (Lj3g3v1790340) and the ABC transporter G family member 36‐like (Lj3g3v2318010), together with three Patellin‐3‐like proteins (Lj3g3v3753340, Lj3g3v3753370, Lj1g3v1381010). The role of both patellins and ABC transporter G family members have been previously linked with virus and non‐host resistance (Song et al., 2020). Interestingly, the enhanced disease susceptibility 1 protein (EDS1‐2; Lj1g3v0416380) was uniquely accumulated in B+ Myc versus NoMyc comparison. EDS1 triggers early plant defenses acting redundantly with salicylic acid to regulate resistance gene‐mediated signaling (Vanugopal et al., 2009).

Our data reveal that *G. margarita* triggers the activation of genes dealing with plant response to biotic and abiotic stress, such as in other AMF. Looking at the individual transcripts of the pathogen responses, both the fungal lines activate the key steps of the PTI pathway, but some plant transcripts and proteins seem to be exclusive of the responses elicited by the B+ fungal line.

### Phenylpropanoid metabolism is finely modulated by the AMF

Mycorrhizal colonization by *G. margarita* modulated phenylpropanoid biosynthesis (lja00940), which is related to coumarin and lignin production (Figure S7). We observed a general upregulation of genes coding for key enzymes in the upstream lignin biosynthetic pathway such as 4‐coumarate‐coenzyme A (4‐coumarate‐CoA) ligase (E.C.6.2.1.12), and in the parallel coumarin pathway (beta‐glucosidases, E.C. 3.2.1.21). Downstream genes coding for caffeoyl‐CoA *O*‐methyltransferase (E.C.2.1.1.104) and caffeic acid *O*‐methyltransferase (E.C.2.1.1.68) were, by contrast, downregulated. These two methyltransferases may modulate lignin content and composition being directly involved in the synthesis of p‐coumaryl, coniferyl, and sinapyl alcohol monomers, which are the building blocks of lignin polymer (Xie et al., 2019).

The involvement of the lignin pathway upon mycorrhization has already been described in many plant models, including tomato (Chialva et al., 2018; Rivero et al., 2015), grape (Bruisson et al., 2016), and *Medicago* (Adolfsson et al., 2017) as well as woody species with secondary growth, such as poplar (Liu et al., 2014) and willow (Aliferis et al., 2015). To confirm that the two lines of *G. margarita* may elicit lignification in mycorrhizal roots, an experiment of lignin quantification was developed. However, the mycorrhization system we adopted for the RNA‐seq on *L. japonicus* imposes an early plant growth stage, and produces a limited amount of material, hampering lignin detection with classical colorimetric methods. For this experiment, we translated the *G. margarita* B+ and B– mycorrhizal systems to another legume plant, *Trifolium repens*. Thanks to this easy‐to‐grow model we performed colorimetric lignin quantification in both roots and shoots. Results showed that lignin significantly increased in roots after mycorrhization (Figure S8, *P* < 0.05), irrespective of the presence of endobacteria into the fungal symbiont. This trend was also observed at systemic level in the shoot even if with a weaker statistical support. To understand whether the lignin deposition involved colonized cells, roots from the same plants were stained with fluoroglucinol. Lignin was detected exclusively in the xylem elements, while no lignin deposition was observed in the epidermal layer or in the arbusculated cells (Figure S9), differently from the lignin impregnation detected in cell wall appositions (papillae) at fungal entry sites in *Petunia* roots (Chen et al., 2021). Thicker older roots from clover revealed, however, random lignified epidermal cells, which were not associated with fungal entry points.

In addition to the response to the mycorrhizal fungus, the datasets revealed a fine regulation of the early steps of phenylpropanoid metabolism, when comparing B+ Myc and B− Myc with the NoMyc condition. The pathway was in fact enriched in the B− Myc versus NoMyc comparison with 54 DEGs, while 36 DEGs emerged from the B+ Myc versus NoMyc contrast with a comparable expression pattern (Figure S7). While the above‐illustrated genes revealed a comparable profile, other genes revealed a different expression pattern: those coding for cinnamyl‐alcohol dehydrogenase (EC:1.1.1.195) were upregulated only in B− Myc versus NoMyc, while those for hydroxycinnamoyl‐CoA transferase (E.C.2.3.1.133) were downregulated in B+ Myc versus NoMyc. A Laccase 1‐like gene (Lj4g3v3114580, LAC1), which has been characterized in Arabidopsis as involved in G‐lignin (guaiacyl lignin unit) synthesis and lignification of xylem fibers (He et al., 2019) was upregulated in both B+ Myc and B− Myc versus NoMyc contrasts.

Proteomics confirmed a differential amount of proteins involved in lignin metabolism which are modulated by mycorrhization and by the endobacterium presence. Three dirigent‐like protein (Lj0g3v0150409 Lj3g3v0821380, Lj3g3v0821330), which are part of the machinery that builds up extracellular lignin‐based structures (Paniagua et al., 2017), decreased in B+ Myc versus NoMyc comparison. Dirigent‐like proteins are extracellular glycoproteins present in all land plants, which have been suggested to play a role in the targeting of coniferyl alcohol to the lignification initiation sites. Differently from the transcriptomic dataset, where only one class III plant peroxidase (E.C.1.11.1.17) was differentially regulated, proteomic analysis showed that nine of 13 peroxidases and peroxidase‐like enzymes accumulated in B+ Myc roots (Lj2g3v1728950, Lj0g3v0336949, Lj0g3v0321999, Lj4g3v0338480, Lj4g3v2172790, Lj4g3v2951200, Lj6g3v1665360, Lj5g3v1927330, Lj3g3v1340390), while no regulation was observed in B− Myc roots. The accumulation of several extracellular peroxidases and peroxidase‐like enzymes (Lj0g3v0336949, Lj3g3v1340390, Lj0g3v0321999, Lj5g3v1925220) was also detected in the B+ Myc versus B− Myc comparison.

Overall, the dataset suggests an upregulation of the phenylpropanoid pathway in mycorrhizal roots, starting from p‐coumaric acid, which is the precursor of all lignin monomers. The results reveal, however, a fine modulation of specific key enzymes. On the one hand, activation of 4‐coumarate‐CoA ligase probably reflects an ongoing lignification due to the young developmental stage of the sampled plants (Cheng et al., 2013; Wang et al., 2016), and on the other, the downregulation of genes and enzymes directly involved in the synthesis of sinapyl (S) and coniferyl (G) alcohols as well as the activation of LAC1, suggest that AM symbiosis can not only modulate the overall lignin content (Rivero et al., 2015), but also the tuning of the lignin S/G monomers ratio. Previous studies have also suggested that similar alterations do not impair plant fitness or produced biomass, but have an effect on lignin architecture, favorably affecting traits such as digestibility for animals (Tu et al., 2010). In addition to the mycorrhization effect, both transcriptomics and proteomics also revealed a potential endobacterial impact, probably inducing a further fine regulation of lignin monomer composition.

On the whole, the data highlight that at the early stages of the colonization process, *G. margarita* elicits key enzymes involved in the upstream phenylpropanoid pathway, which leads to a greater amount of lignin at the later colonization stages, as seen in *T. repens* mycorrhizal roots.

The evolutionary acquisition of the phenylpropanoid pathway is ancestral and dates back to charophytic algae before land colonization (Tohge et al., 2013). In the early divergent liverwort *Marchantia polymorpha*, the pathway is regulated when the thallus is attacked by an oomycete pathogen, similar to what happens in model angiosperms (Carella et al., 2019). The induction of the phenylpropanoid pathway is considered a rather conserved trait with a double role: the structural one, which allows plants to transport water from soil to the leaves providing a strong mechanical support, as well as a response to pathogenic and beneficial microbes. The synthesis of monomers with antimicrobial activity and of the lignin polymers as a cell wall barrier is a defense against pathogens (Lee et al., 2019), and a priming mechanism activated by beneficial microbes, such as plant growth‐promoting bacteria (Safdarian et al., 2019). In addition, the exudation of some coumarins promotes the recruitment of beneficial microbes, including AMF (Cosme et al., 2021; Stringlis et al., 2018). The results obtained here suggest a double role of the phenylpropanoid pathway upon mycorrhization. On the one hand, the signals from beneficial fungi may elicit the biosynthesis of lignin, which remains localized in the cell walls of vascular cells of mycorrhizal roots, holding a structural role that could contribute to the mycorrhizal growth effect, i.e. the iconic result of the AM symbiosis at systemic level. On the other hand, lignin biosynthesis could also be involved in the improved defense response classically displayed by mycorrhizal plants.

### FA metabolism is activated in *Lotus japonicus* mycorrhizal roots with an endobacterium‐dependent modulation

Lipid metabolism in *L. japonicus* was deeply affected by mycorrhization. In addition to an overall increased expression of the phytosterols pathway (lja00100; Figure S10), we found FA‐related categories to be enriched and upregulated in both B+ and B– versus NoMyc comparisons (Figure 3b). AMFs are dependent on host‐produced lipids (Keymer and Gutjahr, 2018), and therefore lipid transfer from the plant to the symbiont is a marker of fungal accommodation. In plastids of arbusculated cells, the activity of the AM‐induced FatM thioesterase releases the acyl group of the forming FA from the acyl‐carrier protein (ACP; Bravo et al., 2017). The AM‐specific RAM2 transforms the acyl‐group acceptor, 3‐phosphate glycerol, into monoacylglycerol (MAG) thanks to an additional phosphatase domain, and preferentially uses ω‐oxidated FAs. The *sn*‐2 MAGs can be transferred to the AMF symbiont through the periarbuscular membrane, probably thanks to the STR/STR2 complex (Keymer and Gutjahr, 2018).

**Figure 5:**
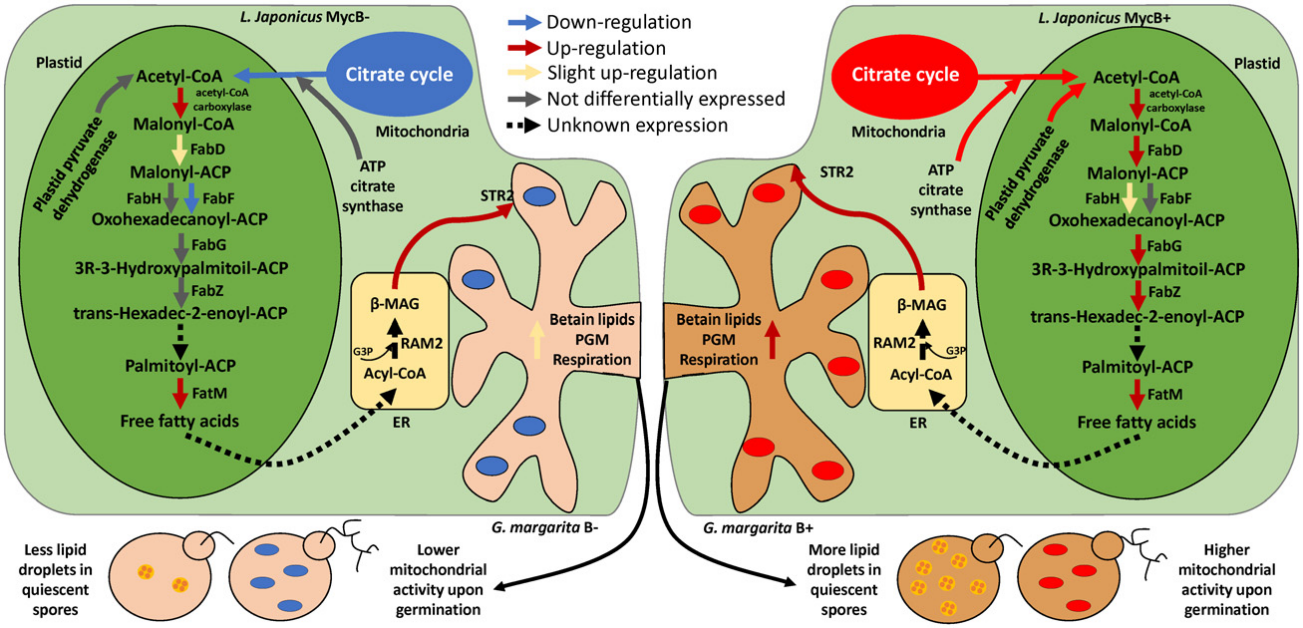
Scheme summarizing the expression of *Lotus japonicus* genes involved in fatty acid metabolism in B– (left) and B+ (right) mycorrhizal roots. Color of the arrows indicates the regulation of genes involved in the pathway in the two comparisons (B− Myc versus NoMyc and B+ Myc versus NoMyc). Dashed lines indicate processes for which a specific gene has not been identified, or which expression is not known according to the present RNA‐seq dataset (such as RAM2). Briefly, while symbiosis‐specific genes, such as FatM and the transporter STR2 seem to be upregulated both in the presence or absence of *Ca*Gg, the upstream production of free fatty acids seems to be only weakly activated in its absence. As citrate cycle, which produces acetyl‐CoA, is upregulated in B+ Myc versus B− Myc, such input required by the pathway may also be reduced in B− Myc cells. One potential effect of a reduced carbon flux from the plant to the B– fungus may be already observable in the intraradical mycelium, where betain‐lipid metabolism and phosphoglycerate mutase (PGM), as well as cellular respiration, are already weakly activated, when compared with the B+ fungus. Such impairment would be reflected on second‐generation spores, which, as already observed (Lumini et al., 2007; Salvioli et al., 2016), have less lipid storage and produce less ATP upon germination. ACP, acyl‐carrier protein; CoA, coenzyme A; MAG, monoacylglycerol.

In *L. japonicus* roots colonized by both the B+ and B− *G. margarita* lines, FatM (Lj5g3v2169500, not mapped in the KEGG database) was strongly upregulated (Figure 5); as RAM2 is apparently missing from the current version of *L. japonicus* gene models (v.3), we could not correctly assess its expression in the context of our RNA‐seq pipeline. However, *L. japonicus* STR/STR2 transporters (Lj4g3v3115140/Lj0g3v0104499), were upregulated in both B+ Myc and B− Myc, indicating that plant–fungus lipid exchange was active with both fungal lines.

While these changes are in line with previous data, some line‐specific responses emerged. FabD, one of the components that initiate the FA biosynthetic pathway, by ligating malonyl‐CoA to the ACP, was only weakly activated in B− Myc, while it had a strong activation in B+ Myc. Several FabG homologs and FabZ (FASII, lja00061 KEGG pathway, Figure S11) acting upstream of FatM, were more expressed in B+ Myc, when compared with B− Myc. FabG (which has several candidates in *L. japonicus*) and FabZ (Lj4g3v0340700) also followed this pattern; always in the FASII pathway, B− Myc roots showed a downregulation of the FabF gene Lj0g3v0190609. In addition, the B– line failed to activate FabH, which synthesizes 3‐oxoacyl‐ACP, and by contrast is upregulated in B+ Myc roots.

Proteomic analysis also revealed the increase of two enzymes involved in lipid biosynthesis in B+ Myc roots. The ATP citrate synthase (Lj4g3v2265020), the primary enzyme responsible for the synthesis of cytosolic acetyl‐CoA, used for the elongation of FAs showed an increased amount in B+ Myc versus B− Myc. The plastid pyruvate dehydrogenase (Lj2g3v1365830), which supplies acetyl‐CoA through its action on pyruvate resulting from glycolysis or the pentose phosphate pathway, was uniquely found in the B+ Myc versus NoMyc comparison.

The discoveries from plant–AMF lipid transfer mostly focus on the terminal steps of *sn* **‐**2 MAG biosynthesis (Bravo et al., 2017): our data reveal the presence of AM‐modulated acyl‐ACP transforming activities, which act upstream of FatM in the plastids. According to our data we can conclude that, while still inducing the export of *sn* **‐**2 MAGs by the host plant, the impact of the B– line on the host FA biosynthetic activities is of lower magnitude, when compared with the B+. We previously demonstrated that the absence of the *Ca*Gg endobacterium strongly affects the lipid reservoir in *G. margarita* spores (Lumini et al., 2007), leading to a different lipid profile (Salvioli et al., 2010). Our new datasets allow us to detect the molecular events, which might result in a limited plant–fungus lipid transfer and then to a lower lipid accumulation in *G. margarita* B– spores. Indeed, this putative differential plant–fungus lipid transfer postulated in the comparison between B+ and B– Myc roots seems to be reflected in the fungal transcriptome (Data S2), as a betaine lipid‐related protein (g13593) is upregulated in the B+ symbiotic mycelium, together with the phosphoglycerate mutase (g11204), which has 3PG as substrate.

The alteration of lipid metabolism observed in the B– Myc plants, although it does not affect the fungal capacity to colonize the plant cells (Lumini et al., 2007), may explain the B– fungal limited capacity in storing lipids.

### Presence of the endobacterium impacts mitochondrial activity and antioxidant processes in the symbiotic roots of *Lotus japonicus*

The previous analyses revealed that GO and KEGG pathways enriched terms largely overlap between either B+ Myc and B− Myc versus the non‐mycorrhizal control, even if many genes relevant for mycorrhization were differentially expressed following the colonization with the two isogenic lines.

However, a reduced number of genes/proteins (151 DEGs, and 119 proteins; Data S1 and 3) were differentially expressed in the direct B+ Myc versus B− Myc comparison, and several of them were involved in mitochondrial activity. Specifically, the NADH‐UQ dehydrogenase subunit 3 was the plant gene most upregulated in B+ Myc in this direct comparison, followed by ATPase 1, several cytochrome *c*‐related genes, and a mitochondrial maturase. Similarly, several proteins of the mitochondrial oxidative phosphorylation pathway such as four ATP synthase subunits (Lj5g3v1853030, Ljchlorog3v0000470, Lj0g3v0054299, Lj0g3v0054299), the NADH dehydrogenase (Lj6g3v0409410) had a higher amount in B+ Myc roots, while the cytochrome *b*–*c*1 complex subunit 7 (Lj1g3v4804100) was exclusively detected in these roots. The tricarboxylic acid cycle was an upregulated biological process in B+ Myc roots with a higher transcript abundance of aconitase (Lj0g3v0329499), isocitrate dehydrogenase (Lj1g3v0488980), succinyl‐CoA ligase beta subunit (Lj5g3v1118370), fumarate hydratase 1 (Lj5g3v1602390), and malate dehydrogenase (Lj3g3v0428550).

Antioxidant processes are required to maintain cellular homeostasis under intense mitochondrial activity, as ROS are produced (Huang et al., 2016). Indeed, two elements that participate in antioxidant processes, a manganese superoxide dismutase and a glutathione *S*‐transferase, were more expressed in B+ Myc roots at transcript level.

Proteomics revealed that two mitochondrial chaperonins CPn60 (Lj0g3v0215859, Lj5g3v1697130) were also more abundant in B+ Myc with respect to B− Myc roots. CPn60 proteins are implicated in the mitochondrial protein import and an intense mitochondrial activity may require their presence to cope with the increase in imported proteins and oxidative stress (Flores‐Pérez and Jarvis, 2013). An increase in abundance of two cytosolic CuZn‐superoxide dismutase (Lj1g3v4955350; Lj1g3v0318300) and a glutathione peroxidase (Lj4g3v2951200), key components of the cellular ROS scavenging system (Bela et al., 2015; Xu et al., 2014) was observed only in the presence of the endobacterium.

In addition, in B+ Myc roots there was the accumulation of two plastid heat shock proteins (Lj6g3v0704480, Lj1g3v4865370), which play a critical role in maintaining the cellular homeostasis under normal plant life or during stress conditions (Aghaie and Tafreshi, 2020).

Looking at the fungal side, the B+ fungus showed an upregulation of two ATP synthase subunits, a NADH‐ubiquinone oxidoreductase, several mitochondrial carriers and glycoproteins, as well as enzymes involved in the Krebs cycle (malate dehydrogenase and citrate synthase), and in ROS detoxification. Three heat shock proteins were also upregulated in the B+ symbiotic mycelium only; these proteins are extremely diversified in AMF, and are thought to be fundamental in maintaining cellular activities under mitochondrial stress (Mothay and Ramesh, 2019).

The OMICs data show that *L. japonicus* B+ Myc plants have a higher antioxidant status when compared with the control and the B− Myc plants, and give molecular support to the observation that carbonylated proteins, which are markers of oxidative stress, increased their amount in *T. repens* and *L. japonicus* after colonization with the cured line (Vannini et al., 2016).

It is well known that AM symbiosis activates antioxidant systems in many crops, particularly under abiotic stress (Bárzana et al., 2015; Zhang et al., 2018). Here we demonstrate that this AM‐specific activation is weakened when the endobacterium is missing. This plant scenario recalls the physiological status observed in the pre‐symbiotic phase of the fungal partner, *G. margarita*; the endobacterium presence leads to increased fungal ATP production, detoxification of mitochondrion‐derived ROS, and increased antioxidative responses (Salvioli et al., 2016; Venice et al., 2017). The current dataset reveals that the impact of *Ca*Gg on *G. margarita* cellular respiration is not limited to pre‐symbiotic growth stages, but persists during the symbiotic phase (Data S2) and this may have a consequence on the mitochondrial and antioxidant responses of the host plant.

Taken on the whole, these results demonstrate that the colonization by *G. margarita* induces mitochondrial activity and antioxidant metabolism in *L. japonicus* roots and, notably, this activation also depends on the presence of the endobacterium hosted in the fungus. The higher bioenergetic potential shown by B+ *G. margarita* during the presymbiotic phase (Salvioli et al., 2016) is maintained during the symbiosis. As genes belonging to the same functional categories are activated in *G. margarita* as well as *L. japonicus*, we can hypothesize the presence of underlying signals, which link plant and fungal responses to the presence of endobacterium.

### Conclusions

The use of a tripartite interaction (the plant host and an AMF, which does or does not contain an endobacterium) has given us the unprecedented possibility to demonstrate that *L. japonicus* can perceive not only the signals produced by an AMF inside its tissues, but also the presence of an endobacterial symbiont thriving in the symbiotic fungus. Notwithstanding the fungal reads represent a low percentage when compared with the plant ones (1.75% versus 71.13%, respectively), the AMF activates a high number of plant genes (5236 when considering the different conditions, i.e., about 20% of the *Lotus* genes expressed in the root). On the other hand, the endobacterium, which is present inside the fungal structures in differential amounts (Salvioli et al., 2008), has the capacity to stimulate a considerable number of both plant and fungal genes (151 and 152, respectively).

*Gigaspora margarita*, as the driving force that leads to relevant transcriptomic changes in its plant host, is able to activate most of the symbiotic pathways so far described in mycorrhizal plants: from fungal sensing to membrane trafficking, nutrient transport, ion binding, and plant defense. In addition, activation of the early steps of the phenylpropanoid metabolism suggests that the mycorrhizal fungus elicits the synthesis of lignin monomers, which at the later stages of the development are crosslinked into this strong cell wall macromolecule. The molecular and biochemical results confirm previous data showing an increased lignin content in mycorrhizal roots (Chialva et al., 2018; Rivero et al., 2015), and suggest that the elicitation of such a pathway is not exclusively related to the priming or to the defense, but it could be a positive effect of the fungus on the developmental process of the plant. Eliciting lignin synthesis starting from its monomers could be a key to explain the success of the AM symbiosis in vascular plants.

Transcriptomics and proteomics on this unique biological system have revealed that the endobacterium *Ca*Gg, which lives inside *G. margarita* does not overtly impact the symbiotic capacities of the fungus. All the well‐known symbiotic pathways are activated in *L. japonicus*, irrespective of the endobacterium. However, a fine tuning of both plant and fungal responses occurred. Similar to observations by Chialva et al. (2020) in tomato, we confirmed that the mycorrhiza‐inducible phosphate transporter 4 (*LjPt4*) was more strongly upregulated in B+ Myc versus NoMyc. Our previous experiments demonstrated that the bacterium has a relevant effect on the physiology of its fungal host, in terms of regulated genes, proteins and metabolites during the pre‐symbiotic phase (Salvioli et al., 2016). Here, we demonstrate that these effects are maintained also during the symbiotic phase. When inside the host and in the presence of the bacterium, the fungus activates its mitochondrial machinery, as well as ROS‐scavenging genes. Surprisingly, the corresponding pathways are activated also in the host plant, *L. japonicus*; both transcriptomics and proteomics pointed to an increased mitochondrial activity with an upregulated expression of respiratory enzymes. Similar to the behavior of the B+ fungus during the presymbiotic phase, the B+ Myc plants were revealed to be more equipped against oxidative stress. These results suggest that the endobacterium contributes to the plant redox homeostasis, in analogy to what occurs in the mycorrhizal fungus, where the modulation of the intracellular calcium store was hypothesized to be a potential link between the endobacterium and the fungal mitochondrion responses (Salvioli et al., 2016). In this context, it is exciting to note that also the other energy‐related organelle, the plastid, modulated some important pathways depending on the bacterial presence. AMF acquire lipids from their host plants thanks to a metabolic pathway located inside plastids of the arbusculated cells (Bravo et al., 2017). On the other hand, the cured fungal line possesses more reduced lipid stores (Lumini et al., 2007) with a different lipid profile (Salvioli et al., 2010). Here, we demonstrate that the plastid metabolic pathway leading to the monoacylglycerol, the molecule that is transferred from the plant to the fungus, is less active in the B− Myc plants. We could hypothesize that the plant colonized by the cured line has a lower energetic status, an unbalanced redox status and consequently negative feedback, its lipid synthesis is impacted. In the context of the biological market, the B– fungus could be expected to provide less P to the plant, which on its part sanctions the fungus transferring it less lipids (Figure 5).

Metabolic pathways located in plant mitochondria and plastids seem to be the first targets of the presence/absence of the AM endobacterium. As some of the AMF, which have been used to investigate plant responses to the AM symbiosis, possess endobacteria (including *Funneliformis* sp. and *Diversispora epigea*, Bonfante and Desirò, 2017), it will be crucial to understand whether the contribution of the endobacteria on the fungus and on the plant is a general trait in these inter‐kingdom interactions.

Plant microbiota has a deep impact on plant health improving plant nutrition, immunity, and resistance to pathogens (Trivedi et al., 2020). As a component of plant microbiota, AMF are expected to support their host with all these beneficial services, and indeed OMICs approaches here confirm that many of these positive activities are guaranteed in the interaction between *L. japonicus* and *G. margarita*. Vannini et al. (2021) have demonstrated that the AMF *Funneliformis mosseae* is the driving factor in stimulating such services in wheat when compared with free‐living bacteria such as *Azospirillum* or *Paraburkolderia*. However, the impact on the plant, as well its resistance to a pathogen, strongly changes depending whether the AMF is associated to one or to the other of the bacterial microbes. Moving from a bipartite to a tripartite interaction deeply modifies the plant response both locally and systemically. The results of our work reveal that also an endobacterium living inside the fungus may modulate plant responses, giving further support to the idea that plant–microbial networks may be highly specific and that reductionist investigations are required for the design of synthetic microbial communities for translational applications.

## EXPERIMENTAL PROCEDURES

### Plant material, RNA extraction, and sequencing

*Lotus japonicus* (Regel) K. Larsen (GIFU) mycorrhizal plants were obtained with the “Millipore sandwich” method (Novero et al., 2002). Briefly, the root plants were placed between two cellulose nitrate membranes (pore diameter 0.45 mm) along with 20 *G. margarita* spores with or without the endobacterium for the B+ Myc and B− Myc condition respectively, or without the *G. margarita* spores for the NoMyc condition. Plants were watered with a Long‐Ashton nutrient solution (Hewitt, 1966) at low P (1.6 μm) and sampled after 6 weeks of co‐cultures.

*Trifolium repens* roots for lignin quantification and localization were obtained by in pot culture: 50 *T*. *repens* seeds were placed in a pot filled with sterilized quartz sand, along with at least 150 *G. margarita* spores with or without the endobacterium for the B+ Myc and B− Myc condition respectively, or without the *G. margarita* spores for the NoMyc condition. Pots were watered once a week with a Long‐Ashton nutrient solution at low P (3.2 μm) and sampled after 3 months of co‐culture.

RNA was extracted with the NucleoSpin RNA Plant and Fungi Kit (Macherey‐Nagel, Dueren, Germany), checked for integrity by capillary electrophoresis using an Agilent (Santa Clara, CA, USA) 2100 Bioanalyzer with the Agilent RNA 6000 Nano Kit following manufacturer’s instructions, and sequenced at Macrogen (Seoul, South Korea) with Illumina (San Diego, CA, USA) NovaSeq technology (100 bp, paired‐end) at the depth of 50 M reads pair.

These sequence data have been submitted to the GenBank database under accession number PRJNA751155. *G. margarita* isogenic lines are available upon reasonable request to authors for non‐profit research purposes.

### Bioinformatics

Raw reads were pre‐processed with RQCfilter script from the BBTools suite (https://sourceforge.net/projects/bbmap/) to remove common contaminants in RNA‐seq and adapters/spike‐in sequences. Transcript abundances were calculated with salmon (Patro et al., 2017) on *L. japonicus* MG20 reference transcriptome (v.3, available at http://www.kazusa.or.jp/lotus/index.html), correcting for both G + C and hexamer selection biases. Reads not mapping to the plant transcriptome were further aligned using salmon software on the reference *G. margarita* transcriptome (Venice et al., 2020). Transcript abundances were then imported and statistically summarized at gene level into the DESeq2 pipeline (Love et al., 2014) using the “tximport” function. We used HTSfilter (Rau et al., 2013) to calculate a dataset‐specific expression threshold, below which genes were discarded; this was done as the removal of genes with low expression values across all the biological conditions is demonstrated to reduce strongly the statistical noise, flatten unwanted variance, and improve DEGs prediction accuracy (Love et al., 2014; Rau et al., 2013). Two surrogate variables were detected in the experiment with svaseq (Leek, 2014) and their effects normalized with RUVSeq using the “RUVg” function (Risso et al., 2014). DEGs were finally obtained with DESeq2, using the betaPrior setting for log_2_fold‐change shrinkage. The same pipeline was used to analyze fungal transcripts setting the “fitType” parameter as “local” in DESeq2.

Gene annotations were obtained from *Lotus* Base (Mun et al., 2016), integrating both the genome annotation file (lj_v3.gaf) and the assembly gene annotations v3.0 (Lotusjaponicus_MG20_v3.0_annotations.gff3) using a custom R script.

VPA was performed in R on “regularized log”‐transformed counts matrix obtained in DESeq2 using the “varpart” function in “vegan” R package v2.5‐4 (http://CRAN.R‐ project.org/package=vegan). Presence/absence of endobacteria and AMF were considered as factors. Testable fractions were assessed using permutational analysis of variance (*n* = 999) on redundancy analysis model at *P* < 0.05. DEG profiles in the three comparisons were clustered using Euclidean distances and reported as heatmaps. Functional enrichments were performed with the “GOSEQ” R package v1.30.0 (Young et al., 2010) to account for the transcript length bias. Multiple hypothesis testing correction was applied using a 0.01 FDR cutoff. Gene Ontology mapping was retrieved from the genome annotation file (lj_v3.gaf), while KEGG mapping using the client‐side REST access to KEGG website using “keggLink” function in “KEGGREST” v1.18.1 R package (https://doi.org/10.18129/B9.bioc.KEGGREST). In both cases, functional enrichments were performed considering only expressed genes as universe and excluding from the analysis genes without an assigned category. Results were visualized using the “GOCircle” function in “GOplot” v1.0.2 R package (Walter et al., 2015) sorting categories by decreasing *z*‐score and increasing adjusted *P*‐value. Only categories represented by at least 10 DEGs were considered.

All the analyses were performed in R programming environment v3.4.4, using RStudio server v1.2.1335 (Rstudio Team, 2021). Custom scripts are available upon request to Authors.

### cDNA synthesis and real‐time qPCR analysis

RNA samples were diluted to 200 ng µl^–1^ and DNAse treatment performed using the Turbo DNA‐free^TM^ kit (Ambion, Austin, TX, USA) according to the manufacturer’s instructions. DNA absence was checked in PCR assays using the *L. japonicus* ubiquitin‐10 as reference gene (Lj5g3v2060710.1, Guether et al., 2009). cDNA was synthesized from 250 ng of DNA‐free total RNA using the Superscript^TM^ II Reverse Transcriptase Kit (Invitrogen, Waltham, MA, USA) according to the manufacturer’s instructions. cDNAs were diluted 1:2 for quantitative relative expression analysis (real‐time qPCR).

Reactions were carried out using a Rotor‐Gene Q instrument (QIAGEN, Hilden, Germany) in 15 µl reaction volume (2.25 µl of water, 7.5 µl of Rotor‐Gene SYBR^®^ Green PCR Mastermix, 2.25 µl of 3 µm forward and reverse primers and 0.75 µl of cDNA sample). For each sample (at least three for each condition, to two technical replicates were performed. The PCR cycling program was composed by a holding stage (10 min at 95°C) and 40 cycles of 15 sec at 95°C and 1 min at 60°C. A melting curve was generated at 0.5°C increments of 10 sec each in the range 55–95°C and a continuous fluorescence measurement recorded at the end of each cycle. Primers were designed using the *L. japonicus* MG20 transcriptome (see above), the *G. margarita* transcriptome (Salvioli et al., 2016) and genome (Venice et al., 2020) and the *Ca*Gg genome as reference (Ghignone et al., 2012). Primers were designed using primer3 web software (http://bioinfo.ut.ee/primer3/) and purchased from Metabion International AG (Planegg, Germany) (Table S2). Take‐off (C_q_) and amplification efficiency values were exported using Rotor‐Gene Q software in “comparative quantitation” mode. Relative gene expression values were then calculated using as reference for normalization the *Lotus* ubiquitin gene (Guether et al., 2009), the *G. margarita* elongation factor 1α and the bacterial 23S rRNA gene for *Ca*Gg (Salvioli et al., 2008). Gene expression was reported as normalized relative quantities calculated based on gene‐specific amplification efficiencies according to Pfaffl (2001) and using the sample with the lower *C*_q_ value as calibrator. Statistical analysis was performed on log_2_‐normalized normalized relative quantities using analysis of variance and the Tukey’s post‐hoc test.

### Protein extraction and digestion

Protein extractions from roots were performed, starting from 1 g of fresh material. Finely ground samples were suspended in 2.5 ml of extraction buffer (Tris–HCl, 0.5 m, pH 8; sucrose, 0.7 m; NaEDTA, 10 mm; ascorbic acid, 4 mm; β‐mercaptoethanol, 0.4%; phenylmethylsulfonylfluoride, 1 mm; leupeptin, 1 μm; pefabloc, 0.1 mg ml^−1^). An equal volume of Tris‐saturated phenol was added. The samples were mixed and incubated for 30 min at 4°C. The phenol phase was collected after 15 min of centrifugation at 5000 ***g*** at 4°C. Proteins were precipitated overnight with five volumes of ice‐cold 0.1 m ammonium acetate in 100% methanol at −20°C. After 40 min of centrifugation at 17 000 ***g***, the protein pellet was washed twice in 0.1 m ammonium acetate and twice in ice‐cold 80% acetone. The resulting pellets were dried and stored at −80°C until further processing. Three independent protein extractions were performed for each condition tested. An equal amount of proteins was prepared for each biological replication. Protein samples were reduced with 10 mm dithiothreitol, alkylated with 55 mm iodoacetamide, digested using sequencing grade trypsin (Promega, Madison, WI, USA) at a ratio of 1:10 (w/w) for 12 h at 37°C.

### Liquid chromatography tandem mass spectrometry analysis

Liquid chromatography tandem mass spectrometry (LC‐MS/MS) analysis was performed using an EASY‐nLC^TM^ capillary system (ThermoFisher Scientific, San Jose, CA, USA), coupled to an LTQ‐Orbitrap XL^TM^ hybrid mass spectrometer (ThermoFisher Scientific). Sample concentration and desalting were performed online using a column (180 μm × 20 mm; packed with 5‐μm, 100‐Å‐pore‐size Symmetry C18 material; ThermoFisher Corp.) at a flow rate of 15 μl min^−1^ for 1 min. Separation was accomplished on a capillary column (100 μm × 100 mm; packed with 1.7‐μm, 130‐Å pore size bridged ethyl hybrid C18 material; ThermoFisher Corp.). A linear gradient of A and B buffers (buffer A, 3% acetone–0.1% formic acid; buffer B, 97% acetone–0.1% formic acid) from 7% to 45% buffer B over 124 min was used at a flow rate of 0.5 μl min^−1^ to elute peptides into the mass spectrometer. Columns were washed and re‐equilibrated between LC‐MS/MS experiments. Electrospray ionization was carried out at 1.7 kV, with the LTQ‐heated capillary set to 150°C.

Peptide analysis was performed using the data‐dependent acquisition of one MS scan followed by CID fragmentation of the five most abundant ions. For the MS/MS experiment, we selected the three most abundant precursors and subjected them to sequential CID‐MS/MS acquisitions. For the MS scans, the scan range was set to 400–1800 *m*/*z* at a resolution of 60 000, and the automatic gain control (AGC) target was set to 1 × 106. For the MS/MS scans, the resolution was set to 15 000, the AGC target was set to 1 × 105, the precursor isolation width was 2 Da, and the maximum injection time was set to 500 msec. The CID normalized collision energy was 35%; AGC target was set to 1 × 105. Data were acquired by Xcalibur™ software (ThermoFisher Scientific). Each analysis was repeated in triplicate.

### MASCOT identification and label‐free quantification

Acquired MS/MS spectra were transformed into Mascot generic format (.mgf) and used for protein identification, with a licensed version of MASCOT software (http://www.matrixscience.com) version 2.4.0. Proteins identifications were performed using a customized database of *L. japonicus* MG20 (50 596 sequences; 15 288 660 residues originally downloaded from http://www.kazusa.or.jp/lotus/index.html).

The search parameters were as follows: threshold set‐off at 0.05 in the ion‐score cutoff (with 95% confidence); MS/MS fragment ion mass tolerance of ±0.6 Da; enzyme specificity was set to trypsin with one missed cleavage; peptide tolerance was set at 10 ppm; fixed modifications of carbamidomethylation at cysteine (Cys); variable modifications of oxidation at methionine and glutamine as pyroglutamic acid; charge states of peptides were set to +2 and +3. Only peptides with significance scores greater than “identity_ score” were counted as identified. Mascot analyzed three biological replicates; only data with a FDR <5% were used for subsequent data analysis.

Mascot identifications were then processed with Scaffold (Proteome Software Inc., Portland, OR, USA) software. Scaffold software was also used to validate protein identifications derived from MS/MS sequencing results and for label‐free quantification. Scaffold verifies peptide identifications assigned by Mascot using the X!Tandem database searching program (Craig and Beavis, 2003; Searle et al., 2008). Then the software probabilistically validates peptide identifications using peptideprophet (Keller et al., 2002) and derives corresponding protein probabilities using proteinprophet (Nesvizhskii et al., 2003; Searle, 2010).

The Scaffold LFQ default method was used for label‐free relative quantification. This method uses the sum of all the spectra associated with a specific protein within a sample, which includes also those spectra that are shared with other proteins and is referred to as the Total Spectrum Count.

Data obtained were then clustered and heat maps were obtained by XLStat 2016.5 version.

### Microscopical analysis

To evaluate the intraradical colonization, some root segments were stained with cotton blue (methyl blue 0.1% [w/v] in lactic acid 80%), while other were fixed in paraformaldehyde 4% (w/v) in phosphate buffer (50 mm pH 7.2), embedded in agarose 8% and cut in 50 μm thick sections by using a vibratome. Sections were then treated with commercial bleach (10% v/v) for 5 min, rinsed in phosphate buffer and stained for 2 h in a solution 10 mg ml^–1^ of wheat‐germ agglutinin conjugated with tetramethylrhodamine in phosphate buffer. Sections were then analyzed under a confocal microscope (Leica TCS SP2) to detect the arbuscule organization and structure. The 532 nm Ar laser band was used to excite the tetramethylrhodamine and the fluorescent signal was collected at 580 nm.

Some other root segments were processed for ultrastructural analyses as described in Novero et al. (2002). Briefly, root segments were fixed in 2.5% (v/v) glutaraldehyde, postfixed in 1% (w/v) osmium tetroxide, dehydrated in an ethanol series and infiltrated in LR White resin. Thin sections (70 nm thick) were obtained with an Ultracut ultramicrotome (Reichert–Jung, Cambridge, UK) and counterstained with uranyl acetate and lead citrate before being observed under a transmission electron microscope (Philips CM10).

To localize the lignin deposits some clover root segments were sampled, placed on coverslips and stained with a freshly prepared solution of phloroglucinol (25 ml distilled water, 25 ml 100% ethanol, 50 ml HCl 37%, and 2 g of phloroglucinol) for at least 10 min. Samples were then observed under a light microscope (Nikon Eclipse Ci‐L): lignin stains bright cherry red.

### Lignin quantification

For lignin quantification, protein‐free cell wall material was obtained as described in Moreira‐Vilar et al. (2014). Briefly, freeze‐dried root and shoot tissue was ground into powder using a TissueLyser apparatus (QIAGEN) and 50 mg of the resulting material was washed by stirring (20 min) and centrifugation (1600 *g*, 5 min) as following: three times in phosphate‐buffered saline (PBS) (50 mm pH 7.0), three times in 2 ml 1% Triton X‐100 (in PBS), two times in 2 ml 1 m NaCl in PBS, two times in 2 ml dH_2_O and a final wash in 2 ml 100% acetone. After the last wash, purified material was dried for 24 h at 60°C to allow acetone evaporation. The purified cell wall material was then used for lignin quantification using the acetyl‐bromide method (Hatfield et al. 1996), as described in Moreira‐Vilar et al. (2014). Ten milligrams of purified cell‐wall sample was added to 0.5 ml 25% acetyl bromide (v/v in glacial acetic acid) and digested for 30 min at 70°C. Tubes were immediately cooled in melting ice for 2 min and 0.1 ml of 5 m hydroxylamine‐HCl and 0.9 ml of 2 m NaOH added. Samples were transferred in 15 ml tubes, 1:4 diluted with 100% glacial acetic acid and centrifuged 10 min at 2400 *g* to pellet the residual solid phase. Samples were further 1:4 diluted in glacial acetic acid and absorbance at 280 nm measured using 10 mm quartz cuvettes and a Beckam (Brea, CA, USA) DU^®^ 530 UV/VIS spectrophotometer. Absorbance values were interpolated with a standard curve generated (*R* ^2^ > 0.99) with alkali lignin (Merck, Darmstadt, Germany; prod. no. 370959) extract obtained as described above. Results were expressed as mg g^–1^ of purified cell wall material.

## CONFLICT OF INTERESTS

The authors declare that they have no competing interests.

## AUTHOR CONTRIBUTIONS

Conceptualization was initiated by PB, FV, and MC; investigation was done by FV, MC, GD, AC, MN, and PB; formal analysis was performed by FV, MC, SG, AC, and GD; visualization was done by MC, FV, and MN; writing of the original draft was done by PB, FV, MC, and CV, while writing of the review and editing was done by PB, LL, FV, MC, AS, CV, GD, SG, and MN; funding was acquired by LL and PB.

## Supporting information


**Appendix S1**. Additional aspects which characterize the interaction between *Gigaspora margarita* B+/– and its host, *Lotus japonicus*.

**Data S1**. Differentially expressed *L. japonicus* root transcripts (DEGs) in the three analyzed contrasts (FDR <0.05).

**Data S2**. Differentially expressed *G. margarita* transcripts (DEGs) in mycorrhizal B+ versus B– (B+ Myc versus B− Myc) contrast (FDR <0.05).

**Data S3**. Differentially expressed *L. japonicus* root proteins (DEPs) in the three analyzed contrasts (FDR <0.05).

**Data S4**. Gene Ontology (GO) and KEGG pathway functional enrichments (P_adj_ <0.1) of DEGs list in the B− Myc/B+ Myc versus NoMyc contrasts.

**Data S5**. Gene Ontology (GO) and KEGG pathway functional enrichments (P_adj_ <0.1) of DEPs list in the three analyzed contrasts.

**Figure S1**. Ultrastructure of arbusculated cortical cells of *Lotus japonicus* roots when colonized by the AM fungus *Gigaspora margarita* containing (B+ Myc) or not (B− Myc) the endobacterium *Candidatus* Glomeribacter gigasporarum.
**Figure S2**. Experimental setup summary and DEGs numbers obtained in the three analyzed contrasts.
**Figure S3**. Principal Components Analysis plot of *G. margarita* transcriptome containing (B+) or not (B–) its endobacteria in mycorrhizal *L. japonicus* roots.
**Figure S4**. Real‐time PCR relative quantification of *Ca*Gg and *G. margarita* in the tripartite symbiosis system.
**Figure S5**. Ten top functional categories (GOs and KEGG pathways) enriched (*P* < 0.05) among DEPs in *G. margarita* (B+ and B– isogenic lines) mycorrhized *Lotus japonicus* roots versus non‐mycorrhized controls (NoMyc).
**Figure S6**. Plant–pathogen interaction KEGG pathway (ko04626) modulation in B− Myc and B+ Myc *L. japonicus* roots versus the non‐mycorrhizal control (NoMyc).
**Figure S7**. Phenylpropanoid biosynthesis KEGG pathway (ko00940) modulation in B− Myc and B+ Myc *L. japonicus* roots versus the non‐mycorrhizal control (NoMyc).
**Figure S8**. Lignin concentrations, measured using the acetyl‐bromide method, in *Trifolium repens* plants mycorrhized or not with *G. margarita* containing or not *Ca*Gg endobacterium.
**Figure S9**. Fluoroglucinol lignin staining on longitudinal sections of *T. repens* roots colonized by *G. margarita* containing (B+ Myc) or not (B− Myc) *Ca*Gg endobacterium.
**Figure S10**. Steroid biosynthesis KEGG pathway (ko00100) modulation in B− Myc and B+ Myc *L. japonicus* roots versus the non‐mycorrhizal control (NoMyc).
**Figure S11**. Fatty acid biosynthesis KEGG pathway (ko00061) modulation in B− Myc and B+ Myc *L. japonicus* roots versus the non‐mycorrhizal control (NoMyc).
**Table S1**. RNA‐seq library sizes and mapping rate on *L. japonicus* reference transcriptome.
**Table S2**. Real‐time PCR primers used in this study.

## Data Availability

Raw RNA‐seq sequence data have been deposited in the Sequence Read Archive (SRA) of the National Center for Biotechnology Information (NCBI) under accession number PRJNA751155. All other relevant data can be found within the manuscript and the supporting information.
