# Supplementary material for: Symbiotic responses of Lotus japonicus to two isogenic lines of a mycorrhizal fungus differing in the presence/absence of an endobacterium
Source: Plant J. 2021 Dec 6;108(6):1547–64. doi: 10.1111/tpj.15578 (PMC9300078; doi:10.1111/tpj.15578)
Supplement: Supplementary file 1 — Appendix S1. Additional aspects which characterize the interaction between Gigaspora margarita B+/– and its host, Lotus japonicus. [file TPJ-108-1547-s004.pdf]

Appendix S1

Additional aspects which characterize the interaction between *Gigaspora margarita* B+/- and its host, *Lotus japonicus*.

Here, we include a summary of additional aspects which characterize the interaction between *Gigaspora margarita* B+/- and its host *Lotus japonicus.* According to our dataset, the absence of the endobacterium *Candidatus* Glomeribacter gigasporarum (*Ca*Gg) inside the mycelium of *G. margarita* elicits differential plant responses that range from alteration of primary metabolism (ATP production) to weak activation of symbiotic dynamics (such as calcium-related responses, lipid exchange, lignin formation and immunity responses). In addition to these dynamics, we found that specific defence genes are up-regulated by the plant in the absence of the endobacterium. B-Myc plants also show different expression of peptidases that putatively impact arbuscule development, and may alter membrane composition in a pathogen-like response. At the same time, only the B+ symbiotic mycelium seems to activate those enzymes that putatively allow penetration into the host cells (Venice *et al.*, 2020). These aspects are discussed in detail below.

Proteins involved in symbiosis are over-produced in mycorrhizal roots in a *Ca*Gg-dependent manner

Among AM-related categories enriched only in B+Myc vs NoMyc, the proteome data set revealed two GO terms, namely calcium ion binding (GO:0005509) and calcium-dependent phospholipid binding (GO:0005544). These two categories contained three annexins (Lj4g3v2823370, Lj0g3v0166899, Lj0g3v0203419), proteins which are characterized by their ability to reversibly interact with membranes in a calcium-dependent manner, involving for example cytoskeleton rearrangements and regulation of membrane traffic (Lizarbe *et al.*, 2013), and having specific roles in root symbioses (Kustova *et al.*, 2020). The *Medicago truncatula* homologue MtAnn1 has been postulated to have a role in cell cycle reactivation and cytoskeleton rearrangements during the early stages of nodulation (de Carvalho Niebel *et al.*, 1998), while MtAnn2 has been proposed to act in connection with membrane traffic and cytoskeleton rearrangements in arbuscule-containing cells (Manthey *et al.*, 2004). Moreover, only in B+Myc a calreticulin (CRT, Lj5g3v1514670), increased. CRT is an endoplasmic reticulum Ca^{2+} buffering protein playing relevant cellular functions. CRT and Ca^{2+} may regulate the fungal accommodation inside the cortical cells and arbuscule development (Sujkowska-Rybkowska and Znojek, 2018).

Defence-related genes are activated in mycorrhizal *L. japonicus* plants, irrespective of the fungal endobacteria

The GO:0006950 category contains a large number of stress-responsive genes which are indirectly involved in plant immunity. A U-box domain-containing protein 36-like (Lj4g3v1440680) was the gene with the highest differential expression (log_2fold-change>3) within the category in both contrasts, while about 20 different U-box domain-containing genes were present in both B+Myc and B- roots vs the control. As belonging to this family, E3-class ubiquitin ligases were already found as up-regulated upon mycorrhization by *Rhizophagus irregularis* in both *L. japonicus* and tomato (Sugimura and Saito, 2017), as well as involved in abiotic stress responses in legume plants (Song et al. 2017).
A number of universal stress proteins (USPs) were strongly induced upon *G. margarita* colonization being more abundant in the B-Myc line *vs* B+Myc (5 *versus* 2 genes, respectively). These proteins, which are highly conserved through all kingdoms of life and highly expressed under diverse environmental constraints, are largely unknown and probably acts as molecular chaperones being

involved, as demonstrated in *Arabidopsis*, in the interaction with thioredoxin-h1 modulating ROS concentration (Chi *et al.*, 2019).

Regulation of *Lotus japonicus* protein turnover and stability in response to mycorrhization by both *G. margarita* lines

Both transcriptomics and proteomics revealed that protein metabolism is among the most affected by both mycorrhization and *Ca*Gg presence. Among the actors involved in protein stability and maintenance, we found that *Ca*Gg presence led to a greater abundance of two proteins identified as isomerase-like protein precursors (Lj5g3v1601820, Lj1g3v0841310), enzymes involved in the unfolded and refolded protein response that, in the endoplasmic reticulum, catalyze the formation of disulfide bonds that stabilize protein conformations (Takemoto *et al.*, 2002; Onda and Kobori, 2014; Kimura *et al.*, 2015; Lu and Christopher, 2008; Peng *et al.*, 2017). Interestingly, the chaperone activity of the protein disulfide isomerases is regulated by its redox status (Wang *et al.*, 2013).
Additional categories of protein metabolism revealed a specific pattern (Fig. 2a): as in other mycorrhizal plants, *L. japonicus* cysteine peptidases (GO:0008234) were up-regulated in B+ and B- mycorrhizal roots in both transcriptome and proteome; interestingly serine-type carboxypeptidase activity (GO:0004185), containing several serine carboxypeptidase-like proteins (SCPLs) resulted to be mostly up-regulated in MycB+ vs NoMyc (z-score>0). SCPLs are a large family of protein hydrolyzing enzymes that play roles in multiple cellular processes. Their potential role in symbiosis has already been suggested in *Medicago truncatula*, where a tandem Kunitz protease inhibitor (KPI106)-serine carboxypeptidase (SCP1) controls mycorrhiza establishment and arbuscule development (Rech *et al.*, 2013). On one hand, all these peptidases are considered relevant during the dismantle of the arbuscules and the recycling of fungal proteins (Floss *et al.*, 2017); on the other, they seem to be involved in the tolerance to oxidative stress. *OsBISCPL1*-overexpressing plants showed an increased tolerance to oxidative stress and up-regulated expression of oxidative stress-related genes (Liu *et al.*, 2008).

Stigmasterol biosynthesis in *L. japonicus* is influenced by the presence of *Ca*Gg

In our dataset, another finding related to lipid metabolism concerns the plant production of stigmasterol (Supplementary Fig. 8.6). Its biosynthesis is triggered by ROS, achieved through both terpenoids and lipid biosynthetic pathways, and finalized by the activity of the CYP710A1 (EC1.14.19.41), a cytochrome p450 (Griebel and Zeier, 2010). In *A. thaliana*, stigmasterol overproduction, and consequent reduction of the β- sitosterol/stigmasterol ratio in plasma membranes, can be forced by pathogens, favoring their proliferation (Griebel and Zeier, 2010). Two mechanisms, which are presumed to be easily translated to other biological systems, were proposed to explain the role of stigmasterol in pathogen infection: on the one side stigmasterol, when compared to β-sitosterol, is poorly efficient in reducing the membrane permeability upon pathogen attack. On the other side, molecular components involved in plant defense are recruited to motile, sphingolipid-rich platforms, or lipid rafts (Ali *et al.*, 2018); the correct functioning of these components highly depends on the motility of their platforms, which can be significantly reduced by the replacement of β-sitosterol with stigmasterol in the surrounding plasma membrane. In the case of *L. japonicus - G. margarita* system, our dataset indicates that sphingolipid biosynthetic genes were overall induced by both the B+ and B- fungal lines. By contrast, a higher production of stigmasterol was deducted only in B-Myc from the up-regulation of the squalene monoxygenase (EC:1.14.14.17), SMT1 and 2 (EC:2.1.1.143) and CYP710A1.

Putative plant cell-wall degradation processes in B+ and B- symbiotic mycelium

Even if the absence of plant cell wall-degrading enzymes is peculiar of AM fungi genomes, *G. margarita* possesses several expanded gene families of Carbohydrate-Active Enzymes (CAZymes) (Venice *et al.*, 2020). Some of these enzymes, such as extracellular peroxidases, are auxiliary and may indirectly attack plant cell wall polymers through oxidative damage, explaining the fungal ability to penetrate plant roots without triggering hypersensitive response in host cells (Venice *et al.*, 2020). In the current dataset, an extracellular dihydrogeodin oxidase/laccase was found to be markedly activated in the B+ symbiotic mycelium only (Supplementary Table 7). Additionally, three CAZymes that target chitin (CE4 family) were up-regulated in the B+ symbiotic mycelium only. These deacetylases might be as well involved in elusion of plant responses, as deacetylated chitin is not recognized by plant receptors (Gow *et al.*, 2007).

References

Ali, U., Li, H., Wang, X. and Guo, L. (2018) Emerging Roles of Sphingolipid Signaling in Plant Response to Biotic and Abiotic Stresses. *Molecular Plant*, **11**, 1328–1343.

Carvalho Niebel, F. de, Lescure, N., Cullimore, J.V. and Gamas, P. (1998) The Medicago truncatula MtAnn1 Gene Encoding an Annexin Is Induced by Nod Factors and During the Symbiotic Interaction with Rhizobium meliloti. *MPMI*, **11**, 504–513.

Chi, Y.H., Koo, S.S., Oh, H.T., et al. (2019) The Physiological Functions of Universal Stress Proteins and Their Molecular Mechanism to Protect Plants From Environmental Stresses. *Front. Plant Sci.*, **0**. Available at: https://www.frontiersin.org/articles/10.3389/fpls.2019.00750/full [Accessed July 26, 2021].

Floss, D.S., Gomez, S.K., Park, H.-J., MacLean, A.M., Müller, L.M., Bhattarai, K.K., Lévesque-Tremblay, V., Maldonado-Mendoza, I.E. and Harrison, M.J. (2017) A Transcriptional Program for Arbuscule Degeneration during AM Symbiosis Is Regulated by MYB1. *Current Biology*, **27**, 1206–1212.

Gow, N.A.R., Netea, M.G., Munro, C.A., et al. (2007) Immune Recognition of Candida albicans β-glucan by Dectin-1. *The Journal of Infectious Diseases*, **196**, 1565–1571.

Griebel, T. and Zeier, J. (2010) A role for β-sitosterol to stigmasterol conversion in plant–pathogen interactions. *The Plant Journal*, **63**, 254–268.

Kimura, S., Higashino, Y., Kitao, Y., Masuda, T. and Urade, R. (2015) Expression and characterization of protein disulfide isomerase family proteins in bread wheat. *BMC Plant Biol*, **15**, 73.

Kustova, D.V., Владимировна, К.Д., Dolgikh, E.A. and Анатольевна, Д.Е. (2020) Annexins and their role in the control of symbioses development in plants. *Ecological genetics*, **18**, 293–300.

Liu, H., Wang, X., Zhang, H., Yang, Y., Ge, X. and Song, F. (2008) A rice serine carboxypeptidase-like gene OsBISCPL1 is involved in regulation of defense responses against biotic and oxidative stress. *Gene*, **420**, 57–65.

Lizarbe, M.A., Barrasa, J.I., Olmo, N., Gavilanes, F. and Turnay, J. (2013) Annexin-Phospholipid Interactions. Functional Implications. *International Journal of Molecular Sciences*, **14**, 2652–2683.

Lu, D.-P. and Christopher, D.A. (2008) Endoplasmic reticulum stress activates the expression of a sub-group of protein disulfide isomerase genes and AtbZIP60 modulates the response in Arabidopsis thaliana. *Mol Genet Genomics*, **280**, 199–210.

Manthey, K., Krajinski, F., Hohnjec, N., Firnhaber, C., Pühler, A., Perlick, A.M. and Küster, H. (2004) Transcriptome Profiling in Root Nodules and Arbuscular Mycorrhiza Identifies a Collection of Novel Genes Induced During Medicago truncatula Root Endosymbioses. *MPMI*, **17**, 1063–1077.

Onda, Y. and Kobori, Y. (2014) Differential activity of rice protein disulfide isomerase family members for disulfide bond formation and reduction. *FEBS Open Bio*, **4**, 730–734.

Peng, R.-H., Qiu, J., Tian, Y.-S., et al. (2017) Disulfide isomerase-like protein AtPDIL1–2 is a good candidate for trichlorophenol phytodetoxification. *Sci Rep*, **7**, 40130.

Rech, S.S., Heidt, S. and Requena, N. (2013) A tandem Kunitz protease inhibitor (KPI106)–serine carboxypeptidase (SCP1) controls mycorrhiza establishment and arbuscule development in Medicago truncatula. *The Plant Journal*, **75**, 711–725.

Song, J., Mo, X., Yang, H., Yue, L., Song, J., and Mo, B. (2017) The U-box family genes in Medicago truncatula: Key elements in response to salt, cold, and drought stresses. *PloS one*, **12(**8), e0182402.

Sugimura, Y. and Saito, K. (2017) Comparative transcriptome analysis between Solanum lycopersicum L. and Lotus japonicus L. during arbuscular mycorrhizal development. *Soil Science and Plant Nutrition*, **63**, 127–136.

Sujkowska-Rybkowska, M. and Znojek, E. (2018) Localization of calreticulin and calcium ions in mycorrhizal roots of Medicago truncatula in response to aluminum stress. *Journal of Plant Physiology*, **229**, 22–31.

Takemoto, Y., Coughlan, S.J., Okita, T.W., Satoh, H., Ogawa, M. and Kumamaru, T. (2002) The Rice Mutant esp2 Greatly Accumulates the Glutelin Precursor and Deletes the Protein Disulfide Isomerase. *Plant Physiology*, **128**, 1212–1222.

Venice, F., Ghignone, S., Fossalunga, A.S. di, et al. (2020) At the nexus of three kingdoms: the genome of the mycorrhizal fungus Gigaspora margarita provides insights into plant, endobacterial and fungal interactions. *Environmental Microbiology*, **22**, 122–141.

Wang, Chao, Li, W., Ren, J., Fang, J., Ke, H., Gong, W., Feng, W. and Wang, Chih-chen (2013) Structural Insights into the Redox-Regulated Dynamic Conformations of Human Protein Disulfide Isomerase. *Antioxidants & Redox Signaling*, **19**, 36–45.
