# Supplementary material for: Symbiotic responses of Lotus japonicus to two isogenic lines of a mycorrhizal fungus differing in the presence/absence of an endobacterium
Source: Plant J. 2021 Dec 6;108(6):1547–64. doi: 10.1111/tpj.15578 (PMC9300078; doi:10.1111/tpj.15578)
Supplement: Supplementary file 7 — Figure S1. Ultrastructure of arbusculated cortical cells of Lotus japonicus roots when colonized by the AM fungus Gigaspora margarita containing (B+ Myc) or not (B− Myc) the endobacterium Candidatus Glomeribacter gigasporarum. Figure S2. Experimental setup summary and DEGs numbers obtained in the three analyzed contrasts. Figure S3. Principal Components Analysis plot of G. margarita transcriptome containing (B+) or not (B–) its endobacteria in mycorrhizal L. japonicus roots. Figure S4. Real‐time PCR relative quantification of CaGg and G. margarita in the tripartite symbiosis system. Figure S5. Ten top functional categories (GOs and KEGG pathways) enriched (P < 0.05) among DEPs in G. margarita (B+ and B– isogenic lines) mycorrhized Lotus japonicus roots versus non‐mycorrhized controls (NoMyc). Figure S6. Plant–pathogen interaction KEGG pathway (ko04626) modulation in B− Myc and B+ Myc L. japonicus roots versus the non‐mycorrhizal control (NoMyc). Figure S7. Phenylpropanoid biosynthesis KEGG pathway (ko00940) modulation in B− Myc and B+ Myc L. japonicus roots versus the non‐mycorrhizal control (NoMyc). Figure S8. Lignin concentrations, measured using the acetyl‐bromide method, in Trifolium repens plants mycorrhized or not with G. margarita containing or not CaGg endobacterium. Figure S9. Fluoroglucinol lignin staining on longitudinal sections of T. repens roots colonized by G. margarita containing (B+ Myc) or not (B− Myc) CaGg endobacterium. Figure S10. Steroid biosynthesis KEGG pathway (ko00100) modulation in B− Myc and B+ Myc L. japonicus roots versus the non‐mycorrhizal control (NoMyc). Figure S11. Fatty acid biosynthesis KEGG pathway (ko00061) modulation in B− Myc and B+ Myc L. japonicus roots versus the non‐mycorrhizal control (NoMyc). Table S1. RNA‐seq library sizes and mapping rate on L. japonicus reference transcriptome. Table S2. Real‐time PCR primers used in this study. [file TPJ-108-1547-s007.pdf]

Lotus japonicus differentially responds to two isogenic lines of a mycorrhizal fungus only differing for the presence/absence of an endobacterium

Francesco Venice, Matteo Chialva, Guido Domingo, Mara Novero, Andrea Carpentieri, Alessandra Salvioli di Fossalunga, Stefano Ghignone, Angela Amoresano, Candida Vannini, Luisa Lanfranco, Paola Bonfante

Supporting Information

Supporting Figures

Figure S1. Ultrastructure of arbusculated cortical cells of *Lotus japonicus* roots when colonized by the AM fungus *Gigaspora margarita* containing (B+Myc) or not (B-Myc) the endobacterium *Candidatus* Glomeribacter gigasporarum.

Figure S2. Experimental set-up summary and DEGs numbers obtained in the three analysed contrasts.

Figure S3. Principal Component Analysis plot of *G. margarita* transcriptome containing (B+) or not (B-) its endobacteria in mycorrhizal *L. japonicus* roots.

Figure S4. Real-Time PCR relative quantification of *Ca*Gg and *G. margarita* in the tripartite symbiosis system.

Figure S5. Ten top functional categories (GOs and KEGG pathways) enriched ($p<0.05$) among DEPs in *G. margarita* (B+ and B- isogenic lines) mycorrhized *Lotus japonicus* roots *versus* non-mycorrhized controls (NoMyc)

Figure S6. Plant–pathogen interaction KEGG pathway (ko04626) modulation in B-Myc (left box) and B+Myc (right box) *L. japonicus* roots *versus* the non mycorrhizal control (NoMyc).

Figure S7. Phenylpropanoid biosynthesis KEGG pathway (ko00940) modulation in B-Myc (left box) and B+Myc (right box) *L. japonicus* roots *versus* the non mycorrhizal control (NoMyc).

Figure S8. Lignin concentrations, measured using the acetyl-bromide method, in *Trifolium repens* plants mycorrhized or not with *G. margarita* containing or not *Ca*Gg edobacterium.

Figure S9. Fluoroglucinol lignin staining on longitudinal sections of *T. repens* roots colonized by *G. margarita* containing (B+Myc) or not (B-Myc) *Ca*Gg endobacterium.

Figure S10. Steroid biosynthesis KEGG pathway (ko00100) modulation in B-Myc (left box) and B+Myc (right box) *L. japonicus* roots *versus* the non mycorrhizal control (NoMyc).

Figure S11. Fatty acids biosynthesis KEGG pathway (ko00061) modulation in B-Myc (left box) and B+Myc (right box) *L. japonicus* roots *versus* the non mycorrhizal control (NoMyc).

Supporting Tables

Table S1. RNA-seq library sizes and mapping rate on *L. japonicus* reference transcriptome.

Table S2. Real-Time PCR primers used in this study.

Supporting Data

Data S1. Differentially expressed *L. japonicus* root transcripts (DEGs) in the three analysed contrasts (FDR<0.05).

Data S2. Differentially expressed *G. margarita* transcripts (DEGs) in mycorrhizal B+ *versus* B- (B+Myc *vs*. B-Myc) contrast (FDR<0.05).

Data S3. Differentially expressed *L. japonicus* root proteins (DEPs) in the three analysed contrasts (FDR<0.05).

Data S4. Gene Ontology (GO) and KEGG pathway functional enrichments (P_{adj}<0.1) of DEGs list in the B-Myc/B+Myc *vs* NoMyc contrasts.

Data S5. Gene Ontology (GO) and KEGG pathway functional enrichments (P_{adj}<0.1) of DEPs list in the three analysed contrasts.

Supporting Figures

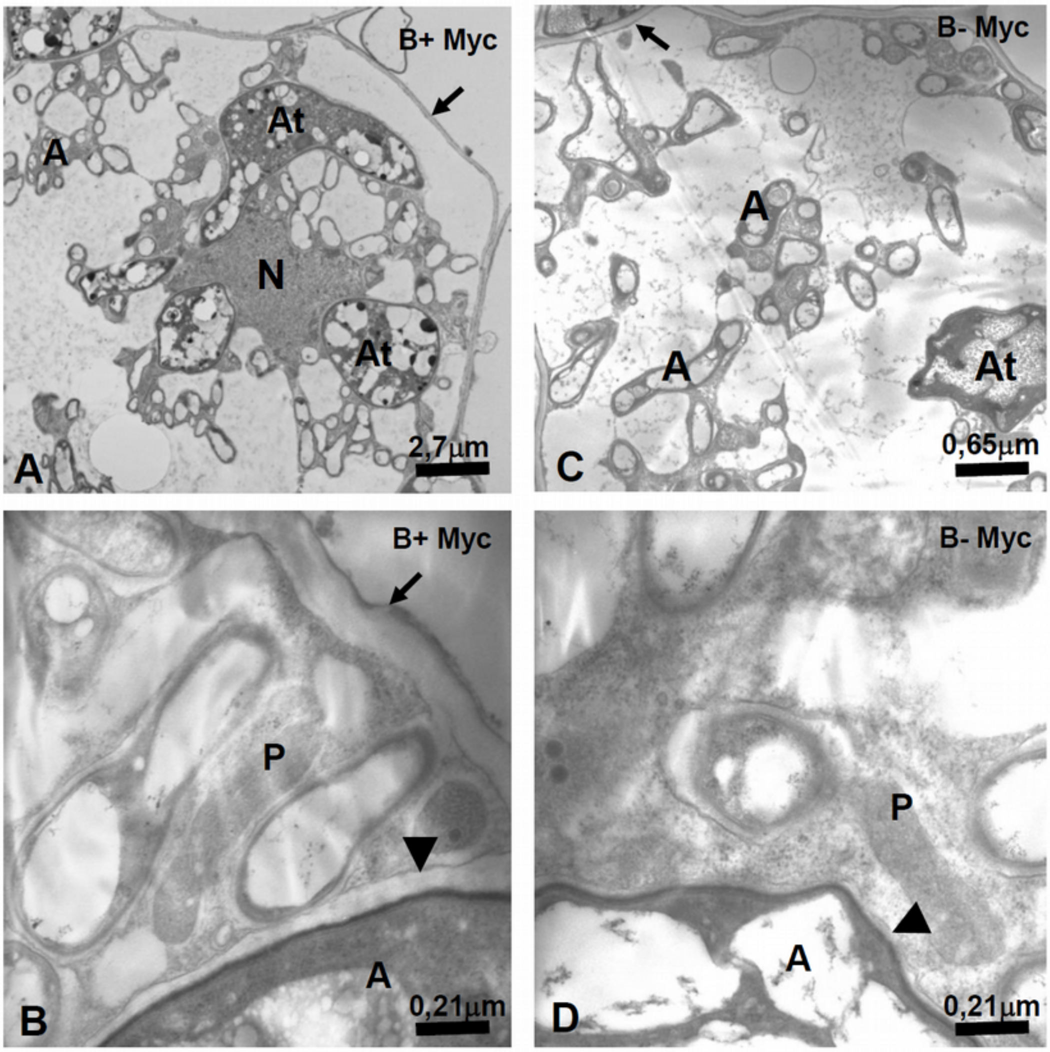

Figure S1. Ultrastructure of arbusculated cortical cells of *Lotus japonicus* roots when colonized by the AM fungus *Gigaspora margarita* containing (B+Myc) or not (B-Myc) the endobacterium *Candidatus* Glomeribacter gigasporarum. A and C illustrate the general organization of a root cortical cell colonized by the arbusculated fungal hyphae. (N: plant nucleus, A: finest arbuscular branches, At: arbuscule main trunks, Arrows: plant cell walls. B and D details of arbuscular branches and host plants plastids. (A: arbuscular branches, P: plastids, Arrow: plant cell wall, Arrowheads: periarbuscular membrane.

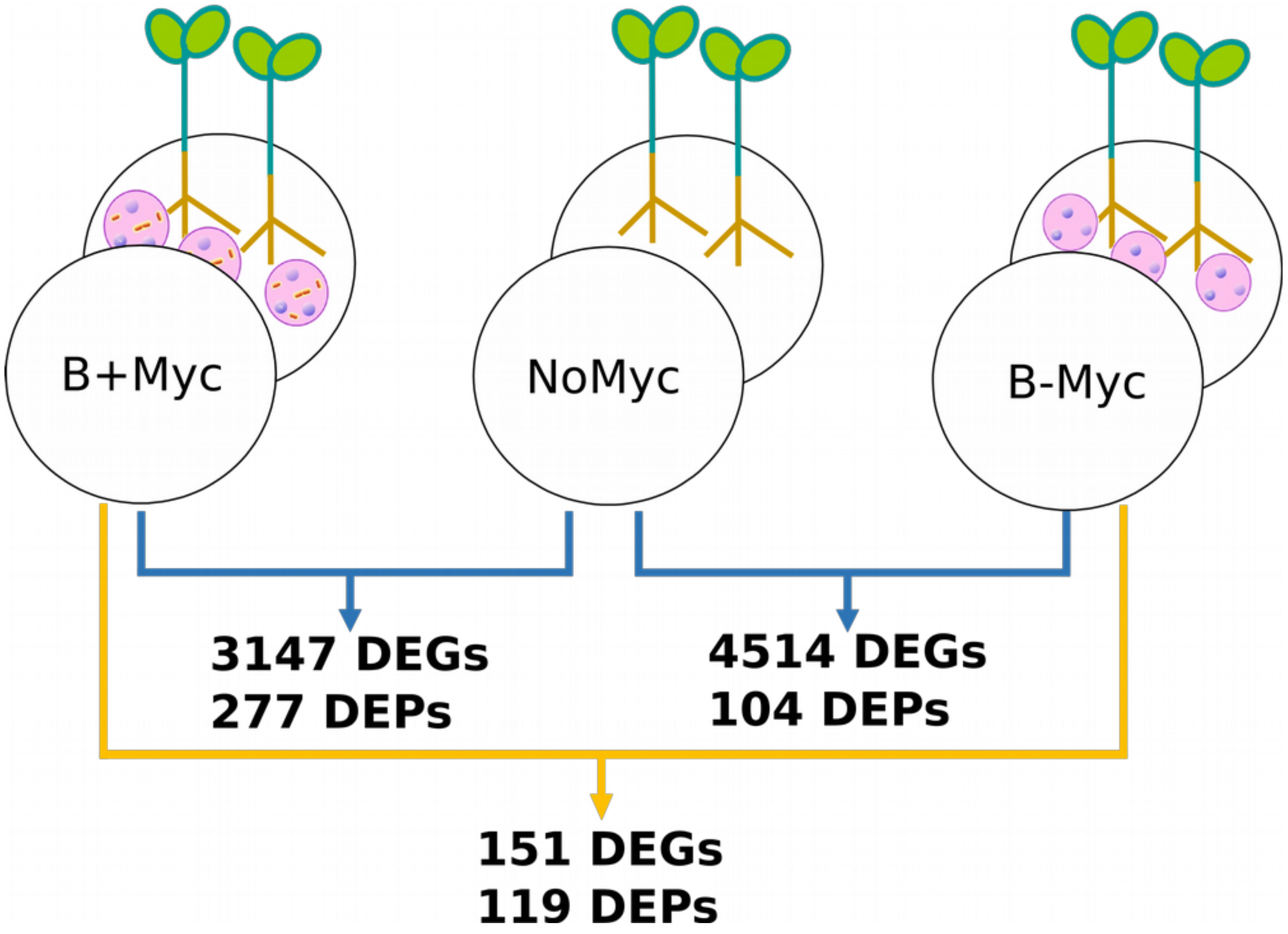

Figure S2. Experimental set-up summary illustrating the three growth condition for *L japonicus* seedlings: Non-mycorrhizal (NoMyc, central position), colonized by *Gigaspora maragarita* with (B+Myc, left side) and without (B-Myc, right side) the endobacterium. The figures refers to the numbers of differentially-expressed genes (DEGs) and differentially expressed proteins (DEPs) revelaed by the transcriptomic and proteomic analysis, respectively.

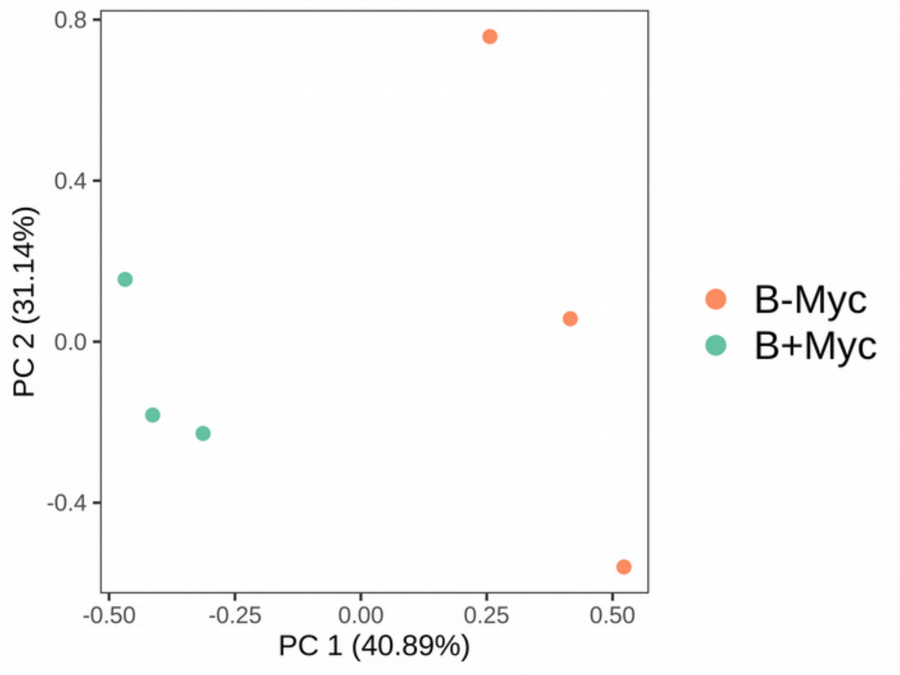

Figure S3. Principal Component Analysis plot of *G. margarita* transcriptome containing (B+) or not (B-) endobacteria in mycorrhizal *L. japonicus* roots. The variance explained by the first two most influential principal components (PC1 and PC12) is shown in brackets.

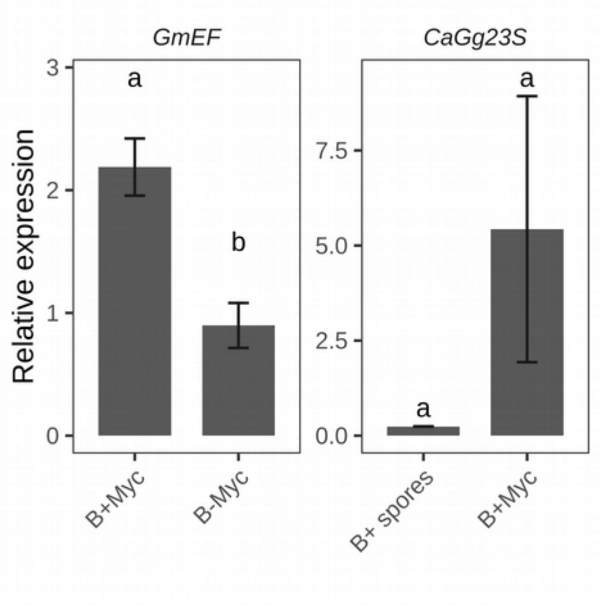

Figure S4. Real-Time PCR relative quantification of *Ca*Gg and *G. margarita* in the tripartite symbiosis system. Relative quantification has been performed normalizing the AM fungus and the *Ca*Gg 23S rRNA gene on the *L. japonicus* ubiquitin and the *G. margarita* elongation factor, respectively. Letters indicate statistically supported differences according to a Welch two-sample t-test ($p<0.05$).

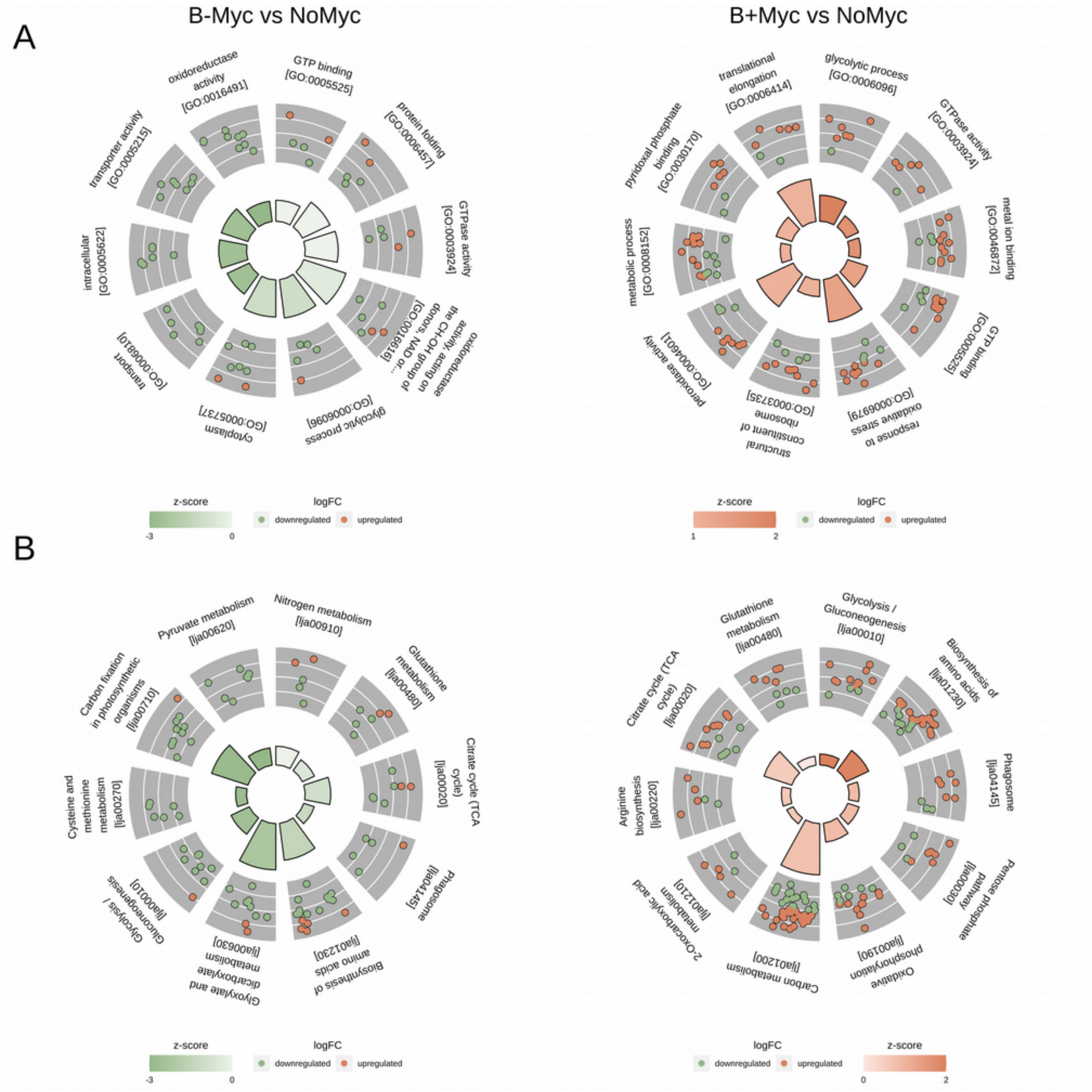

Figure S5. Ten top functional categories (GOs and KEGG pathways) enriched (p<0.05) among DEPs in *L. japonicus* roots colonized by the two isogenic lines of *G. margarita* (B+ and B-) *versus* the non-mycorrhizal control (NoMyc). (A) Gene-Ontology (GO) functional categories; (B) KEGG pathway. Each section of the circle plot represents an enriched term or pathway. The inner barplot plots the - $\log_{10}$ of the adjusted p-value for each enriched term and the colour shows the z-score value, indicating global up- regulation (if >0) or down-regulation (if <0) of genes within each category. Detailed $\log_2$fold-change value of each gene within each category is plotted as dot plot in the outer circle (up-regulated in red and down-regulated in green). The first ten enriched categories were plotted clockwise by decreasing z-score and p-value.

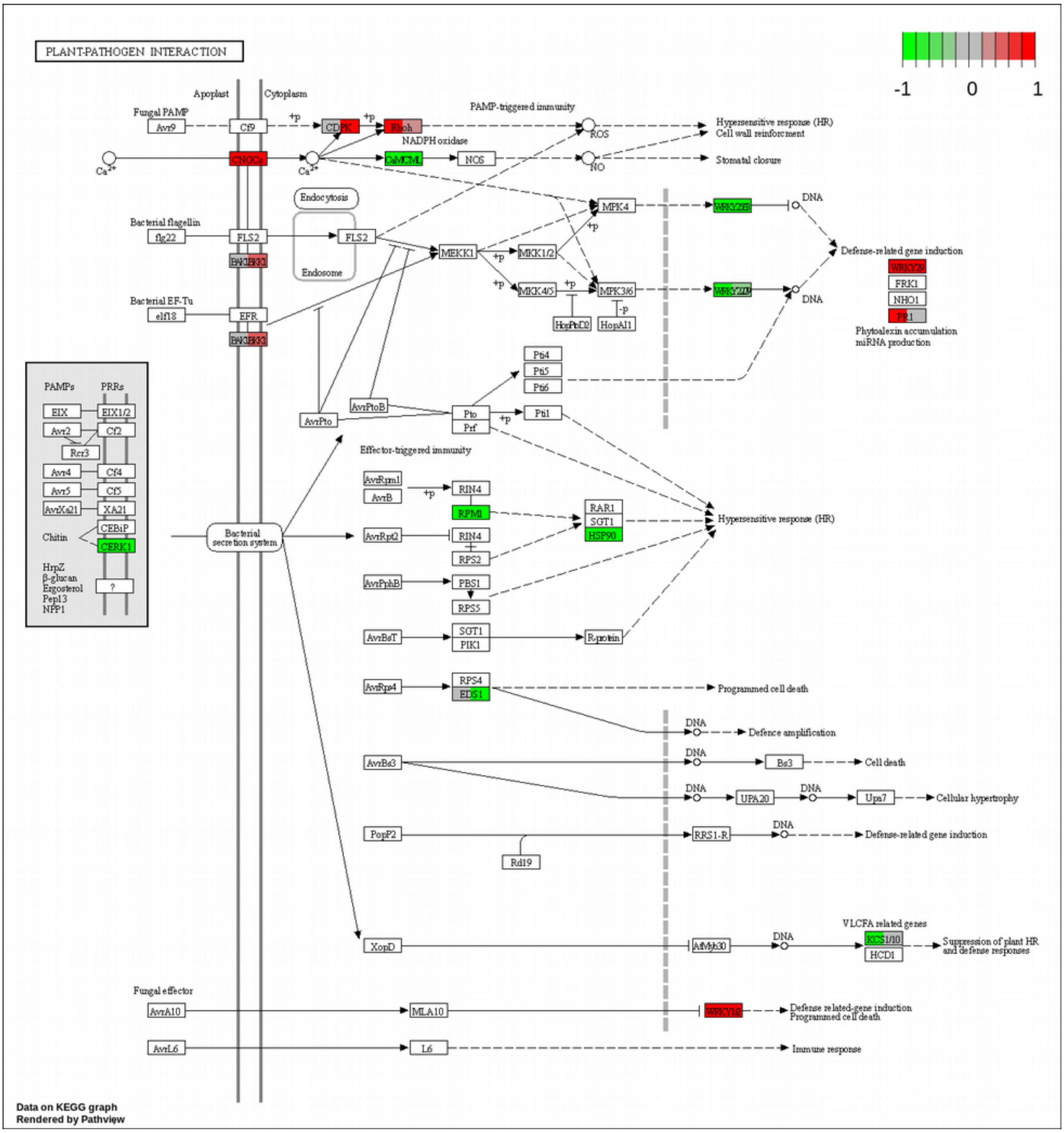

Figure S6. Plant–pathogen interaction KEGG pathway (ko04626) modulation in B-Myc *versus* NoMyc (left box) and B+Myc *versus* NoMyc (right box) *L. japonicus* roots. Up-regulated IDs are displayed in red, down-regulated ones in green.

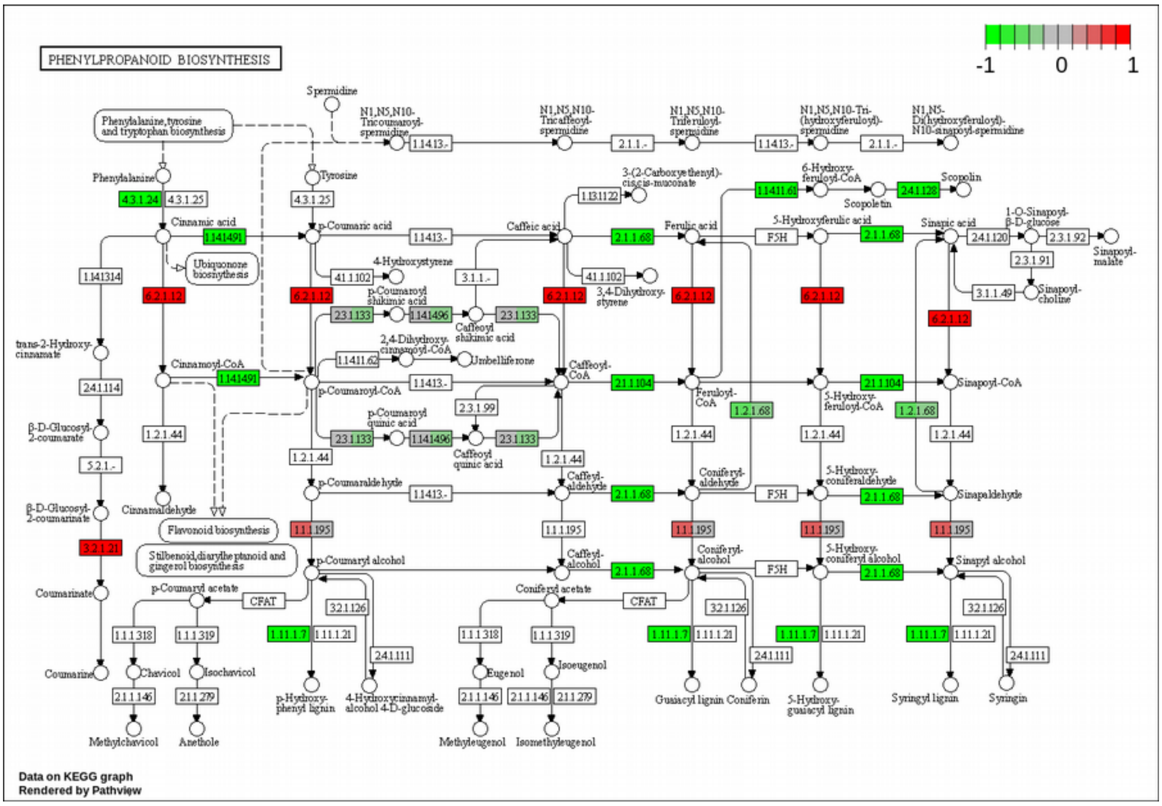

Figure S7. Phenylpropanoid biosynthesis KEGG pathway (ko00940) modulation in B-Myc *versus* NoMyc (left box) and B+Myc *versus* NoMyc (right box) *L. japonicus* roots. Up-regulated IDs are displayed in red, down-regulated ones in green.

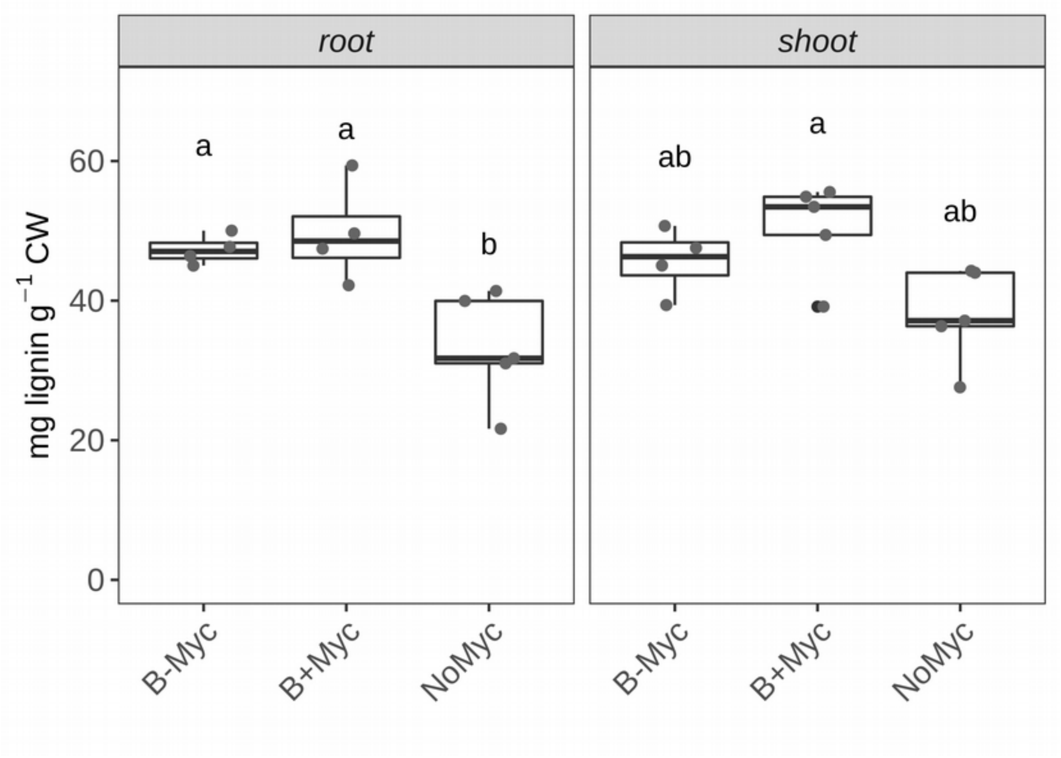

Figure S8. Lignin concentration, measured using the acetyl-bromide method, in *Trifolium repens* plants mycorrhized or not with *G. margarita* containing or not *Ca*Gg endobacterium. Letters indicate significant differences among treatments (ANOVA, Tukey's post hoc test; $P < 0.05$). Lignin amount is expressed as mg per gram of purified cell wall (CW) material; n = 5, boxplots display the median (horizontal line), the quartiles (boxes) and 1.5 interquartile range (whiskers).

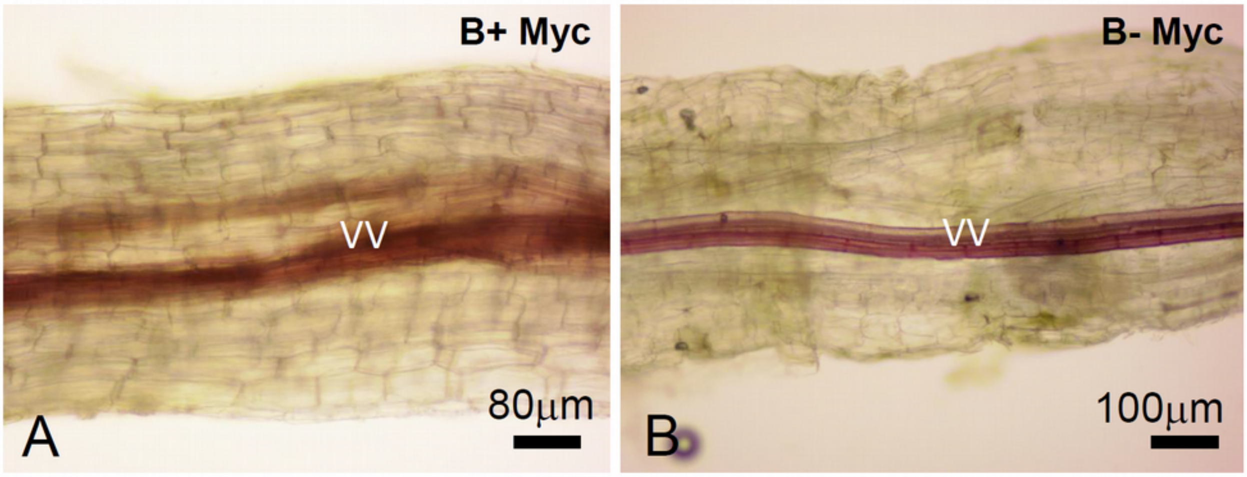

Figure S9. Fluoroglucinol lignin staining on longitudinal sections of *T. repens* roots colonized by *G. margarita* containing (B+Myc) or not (B-Myc) *Ca*Gg endobacterium. A and B overview of the colonized roots (VV: vascular vessels).

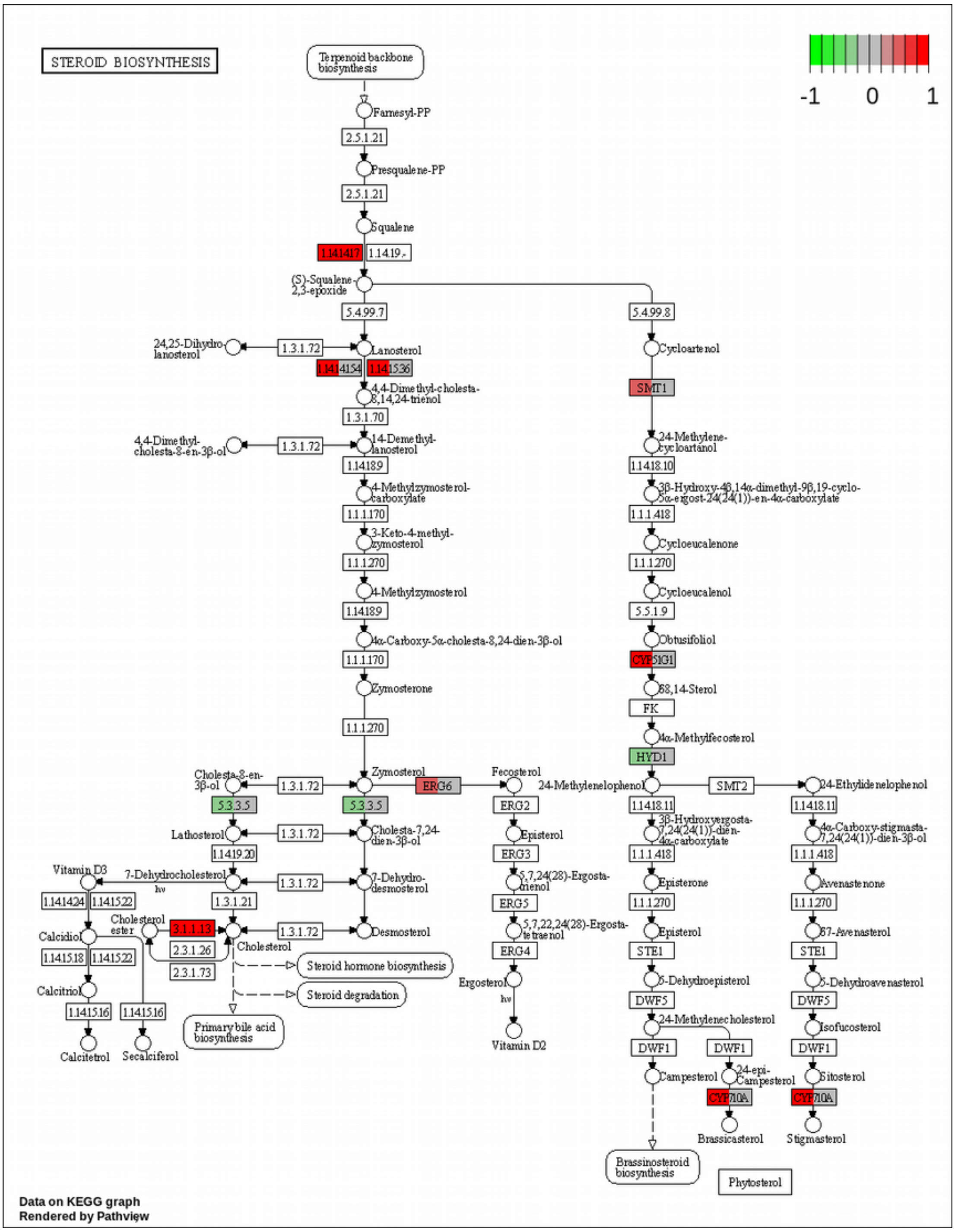

Figure S10. Steroid biosynthesis KEGG pathway (ko00100) modulation in in B-Myc *versus* NoMyc (left box) and B+Myc *versus* NoMyc (right box) *L. japonicus* roots. Up-regulated IDs are displayed in red, down-regulated ones in green.

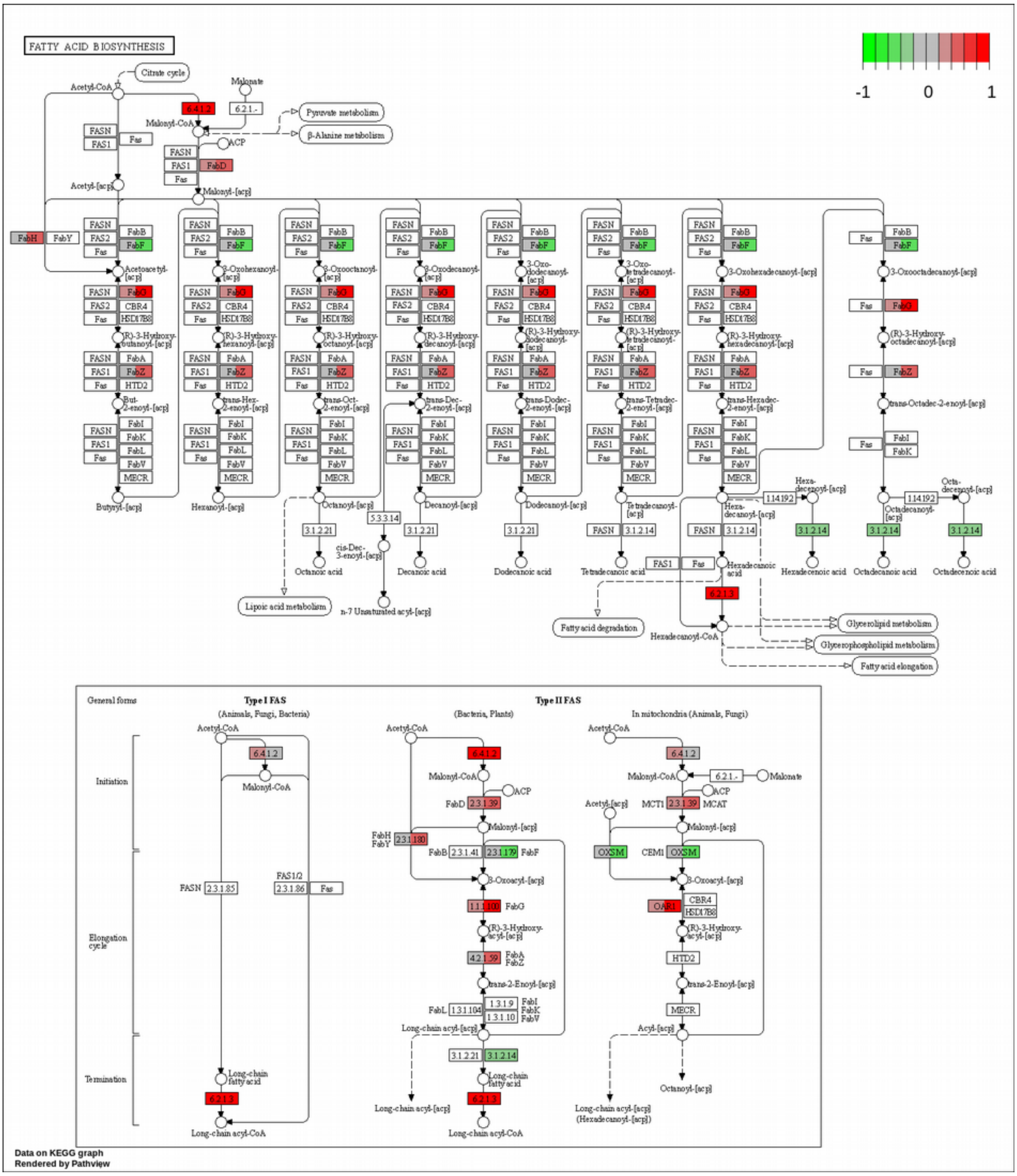

Figure S11. Fatty acids biosynthesis KEGG pathway (ko00061) modulation in B-Myc *versus* NoMyc (left box) and B+Myc *versus* NoMyc (right box) *L. japonicus* roots. Up-regulated IDs are displayed in red, down-regulated ones in green.

Supporting Tables

Library	N. of reads pairs	Mapping rate on *L. japonicus* v.3 transcriptome
NoMyc3	68.404.818	74.87%
NoMyc4	62.219.029	73.38%
NoMyc7	58.509.848	73.40%
B+Myc1	57.932.634	64.94%
B+Myc5	53.826.962	72.82%
B+Myc7	56.652.196	70.95%
B-Myc4	61.271.154	70.50%
B-Myc5	57.444.085	70.86%
B-Myc7	95.349.977	68.43%

Table S1. RNA-seq library sizes and mapping rate on *L. japonicus* reference transcriptome.

Table S2. Oligonucleotides sequences used in this study

Organism	Gene	Annotation	Transcript ID	Forward primer (5' -3')	Revers primer (5' -3')	Reference
Lotus japonicus	*LjUBI*	Ubiquitin-10	Lj5g3v2060710.1	TTCACCTTGTGCTCCGTCTTC	AACAACAGCACACACAGACAATCC	Guether et al., 2009
	LjPT4	AM-induced phosphate transporter 4	Lj1g3v0948470.1	GTACAATGACCTCATGGTTCT	CGTTCATCTCGAAATCCTTATC	Volpe et al., 2013
G. margarita	*EF1a*	Elongation factor 1-alpha	AJ566401	TGAACCTCCAACCAGACCAACTG	CGGTTTCAACACGACCTACAGGGAC	Salvioli et al., 2008
	GmPHO1	Xenotropic and polytropic retrovirus receptor 1	g17792.t1	CGCATTGGATACACCCACCT	CCTTCCTCAGGGCTCGATTG	This study
Ca. Glomeribacter gigasporarum	*CaGg23S*	23S rRNA gene	AJ561042.3	GGGTCCATTGCGGATTACTTC	GTTGTTGCCCTCTTGACACC	Salvioli et al., 2008
	*Ca*GgPT	Phosphate import ATP-binding protein PstB	CAGGBEG34_v5_100026	CCGTTTCCGATGTCGGTGTA	ACGCAGTGTGTTCTTCACCT	This study
